# Yeast Tdh3 (Glyceraldehyde 3-Phosphate Dehydrogenase) Is a Sir2-Interacting Factor That Regulates Transcriptional Silencing and rDNA Recombination

**DOI:** 10.1371/journal.pgen.1003871

**Published:** 2013-10-17

**Authors:** Alison E. Ringel, Rebecca Ryznar, Hannah Picariello, Kuan-lin Huang, Asmitha G. Lazarus, Scott G. Holmes

**Affiliations:** Department of Molecular Biology and Biochemistry, Wesleyan University, Middletown, Connecticut, United States of America; Indiana University, Howard Hughes Medical Institute, United States of America

## Abstract

Sir2 is an NAD^+^-dependent histone deacetylase required to mediate transcriptional silencing and suppress rDNA recombination in budding yeast. We previously identified Tdh3, a glyceraldehyde 3-phosphate dehydrogenase (GAPDH), as a high expression suppressor of the lethality caused by Sir2 overexpression in yeast cells. Here we show that Tdh3 interacts with Sir2, localizes to silent chromatin in a Sir2-dependent manner, and promotes normal silencing at the telomere and rDNA. Characterization of specific *TDH3* alleles suggests that Tdh3's influence on silencing requires nuclear localization but does not correlate with its catalytic activity. Interestingly, a genetic assay suggests that Tdh3, an NAD^+^-binding protein, influences nuclear NAD^+^ levels; we speculate that Tdh3 links nuclear Sir2 with NAD^+^ from the cytoplasm.

## Author Summary

Cells respond to changing signals or environmental conditions by altering the expression of their genes. For instance, our cells respond to the presence of glucose or insulin in the bloodstream by regulating the expression of genes involved in basic cell metabolism. The sirtuin family of proteins has been proposed to serve as a link between a cell's metabolic state and gene expression, although the molecular mechanisms that connect metabolic status with Sir2 activity remain unclear. The expression of genes is controlled in part by the structural organization of the local chromatin region within which they reside. The yeast sirtuin protein, Sir2, mediates repression (“silencing”) of sets of genes by modulating the structural organization of specific chromatin regions. In this study we describe a novel link between a key metabolic enzyme and Sir2 function. We show that a yeast GAPDH protein, which plays a central role in glucose metabolism, also associates with Sir2 in the nucleus and promotes Sir2-dependent gene silencing. Sirtuin activity requires a small molecule, NAD^+^, whose availability may fluctuate depending on the metabolic state of the cell. Based on our data, we suggest that Tdh3 may promote silencing by maintaining sufficient levels of NAD^+^ available to Sir2 within the nucleus.

## Introduction

The yeast Sir2 protein is the founding member of a large family of NAD**^+^**-dependent protein deacetylases (“sirtuins”) conserved among all three domains of life [1], [2]. Yeast Sir2 deacetylates histones, particularly lysine 16 of histone H4, as part of a silencing mechanism that suppresses the transcription of telomere-proximal genes and the silent mating type loci. At these locations, Sir2 acts in conjunction with the Sir3 and Sir4 proteins [3], [4]. Sir2 also acts to reduce recombination and silence expression of RNA polymerase II transcribed genes at the rDNA repeats [5], [6], [7]. Sir2 family members in yeast and other organisms have both histone and non-histone substrates and regulate a variety of cellular processes.

Sir2 and other sirtuins link cleavage of NAD**^+^** to their deacetylation reaction. Sir2's NAD**^+^**-dependence led to the suggestion that it might be regulated by changes in metabolism that affect NAD**^+^** concentrations [2], [8], [9]. In support of this proposal, Sir2-related functions can be affected by manipulating the levels of enzymes in the NAD**^+^** biosynthetic pathway, or by varying the concentrations of NAD**^+^** precursors in the growth media. For example, NAD**^+^** levels are reduced in yeast cells lacking the *NPT1* gene, which codes for a key enzyme in the salvage pathway, reforming NAD**^+^** from nicotinic acid [10]. This drop in NAD**^+^** is accompanied by a decrease in rDNA and telomeric silencing and an increase in rDNA recombination [10]. Addition of the NAD**^+^** precursor nicotinamide riboside restores NAD**^+^** levels in *npt1* mutants and also suppresses their rDNA silencing and recombination defects in a Sir2-dependent manner [11].

In a prior genetic screen for candidate Sir2 regulators we identified Tdh3, a yeast glyceraldehyde 3-phosphate dehydrogenase (GAPDH), which converts NAD^+^ to NADH while executing a key step in glycolysis [12]. Given the links between metabolism, NAD**^+^**, and Sir2 activity, we investigated possible influences of this protein on Sir2. We found that yeast Tdh3 is a Sir2-interacting protein that regulates silencing, influences Sir2's association with chromatin, and modulates nuclear NAD**^+^** levels.

## Results

### Tdh3 regulates transcriptional silencing at the telomere and *HMR* loci

**Figure 1:**
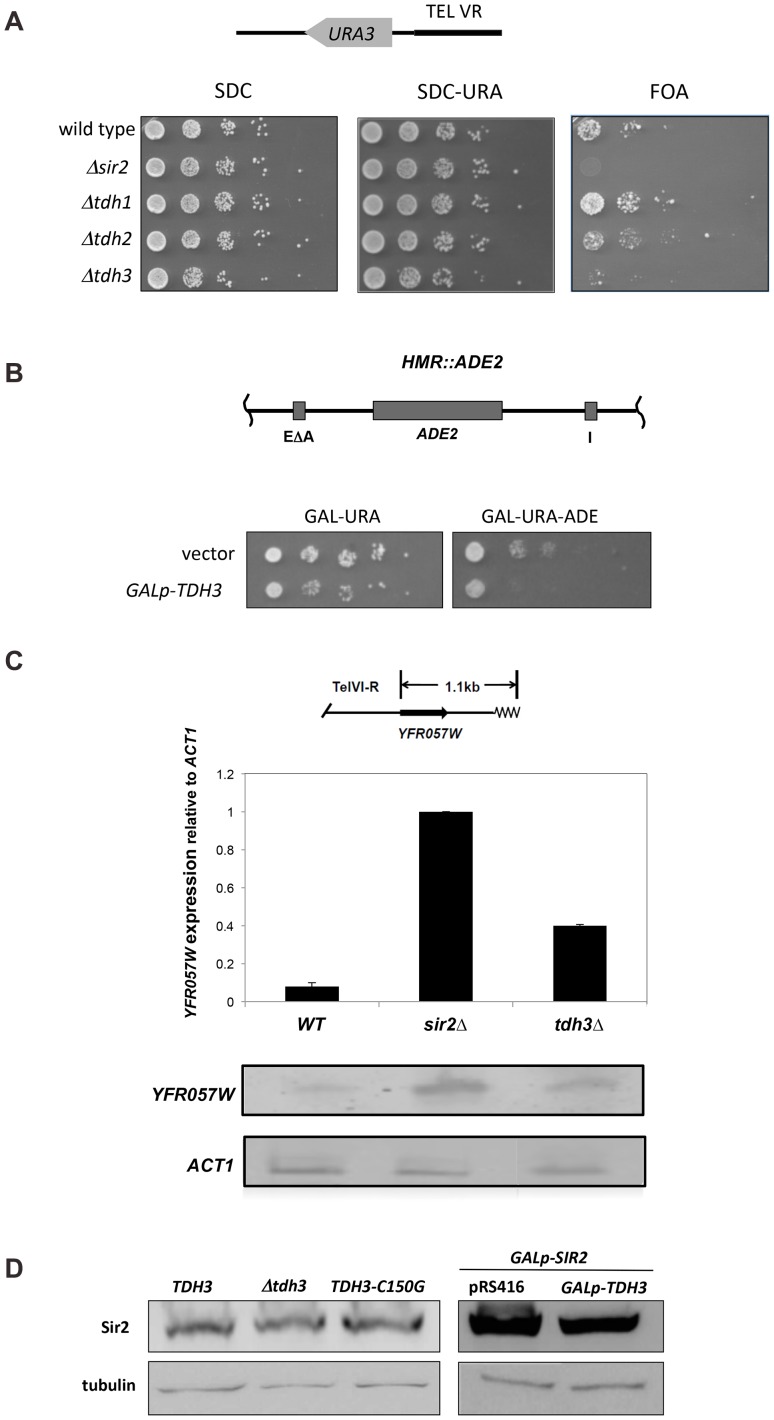
Tdh3 is a novel regulator of Sir2 dependent transcriptional silencing. (A) Tdh3 regulates silencing at the telomeres. Serial dilutions of strains bearing *URA3* reporter gene adjacent to a telomere [17] were made on complete medium (SDC), and on media containing 5-FOA, which counterselects for *URA3* expression. The *URA3* promoter is approximately 1 kb from the telomere repeat sequences [67]. (B) Overexpression of Tdh3 causes an increase in silencing at the *HMR* locus. A plasmid containing the *TDH3* gene fused to the galactose-inducible *GAL1* promoter was introduced into a strain bearing the *ADE2* gene at the *HMR* locus [68]. This strain lacks the Orc binding site at the *HMR-E* silencer (the “A site” of the silencer). Serial dilutions of this stain were grown on the indicated media. (C) Tdh3 regulates expression of an endogenous telomere proximal gene. Expression of the native telomere gene *YFR057W*, was examined by quantitative RT-PCR [60] in the indicated strains. (D) Sir2 protein levels are unchanged in strains lacking or overexpressing Tdh3. Left panel: Sir2 expressed from its endogenous gene was detected via immunoblotting protein extracts made from a wild-type strain, a strain lacking the *TDH3* gene, or a strain expressing a Tdh3 protein with a single amino acid substitution. Right panel: Strains overexpressing Sir2 and bearing either a control vector (pRS416) or a plasmid overexpressing Tdh3 are shown.

There are three GAPDH enzymes in yeast, coded for by the *TDH1*, *TDH2*, and *TDH3* genes [13], 14. Deletion of any one of the three *TDH* genes is not lethal, but elimination of both *TDH2* and *TDH3* causes inviability, indicating these genes have a redundant, essential function [14]; the Tdh1 protein appears to be exclusively expressed in stationary phase [15], [16], and can be deleted in combination with either Tdh2 or Tdh3 without compromising viability. To examine whether GAPDH enzymes influence silencing in yeast we deleted *TDH1*, *TDH2*, or *TDH3* in a strain bearing a *URA3* reporter gene at the telomere [17]. We observed that deletion of *TDH3* caused a decrease in telomeric silencing (Figure 1A). Loss of Tdh1 or Tdh2 did not lead to strong phenotypes in this assay. Since we initially identified *TDH3* by its overexpression phenotype we also determined its influence on silencing when expressed at high levels. We transformed a plasmid containing the *TDH3* gene under the control of *GAL1* promoter into a strain containing the *ADE2* gene integrated at the *HMR* locus. In this assay we find that silencing of the *ADE2* gene is improved in strains overexpressing *TDH3* (Figure 1B).

Since phenotypic assays based on the *URA3* reporter gene may in some cases be subject to influences independent of transcriptional silencing [18], [19], we also examined Tdh3's influence on the transcription of a naturally occurring telomere-linked gene, *YFR057W* (Figure 1C) [20]. An increase in *YFR057W*'s mRNA levels in strains lacking Sir2 indicates that this gene is subject to Sir-dependent silencing (Figure 1C). We observed that loss of Tdh3 caused a significant increase in the expression of this gene, consistent with a role for Tdh3 in mediating telomere position effect. Control experiments indicated that deletion or overexpression of Tdh3 did not alter Sir2 levels in the cell (Figure 1D).

### Tdh3 regulates recombination at the rDNA repeats

**Figure 2:**
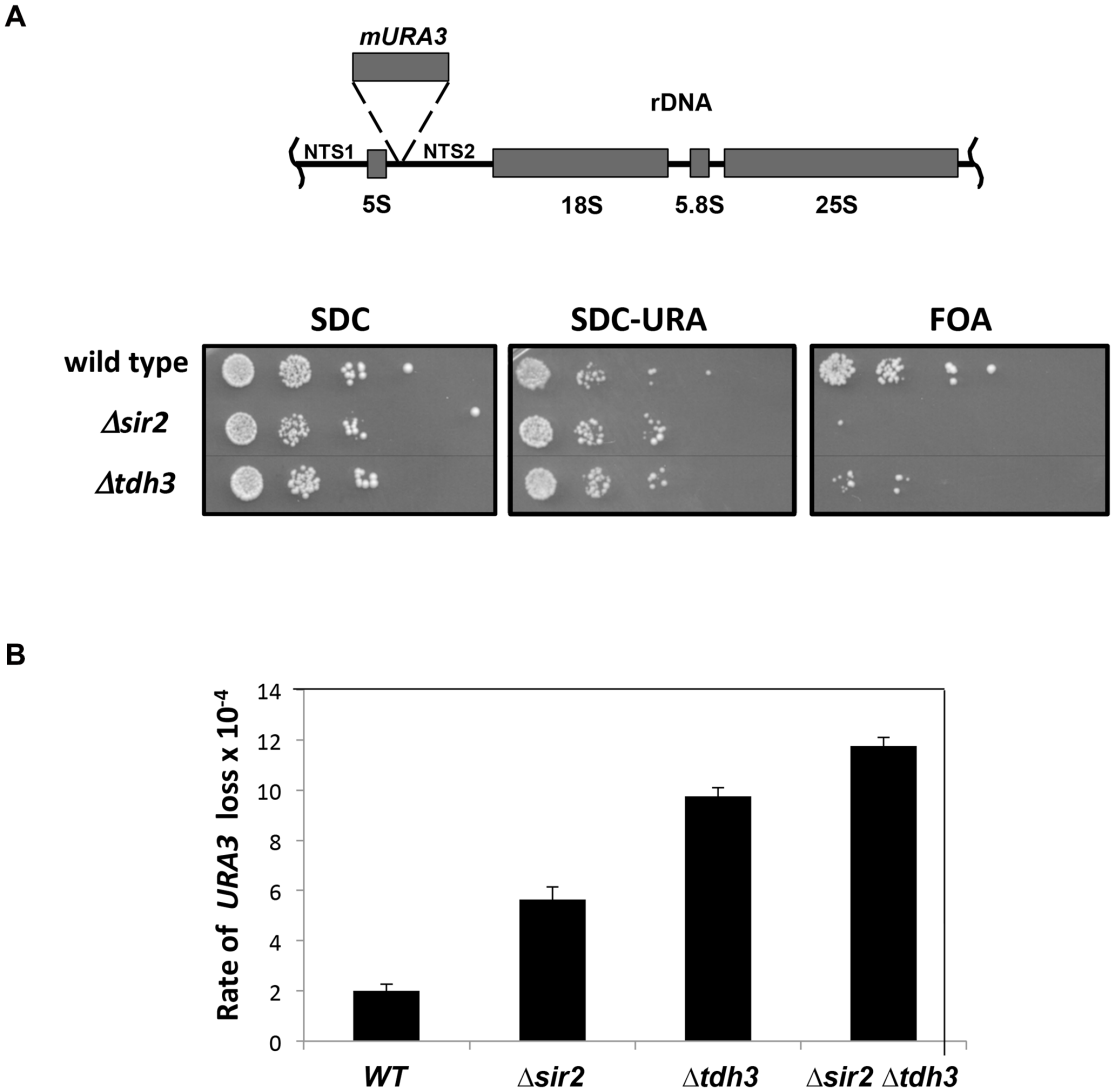
Tdh3 regulates silencing and recombination at the rDNA repeats. (A) Tdh3 regulates silencing at the rDNA locus. Serial dilutions of strains bearing the *mURA3* reporter gene embedded in the non-transcribed spacer (NTS) region of the rDNA repeats were made on the indicated media. *mURA3* has a compromised promoter, and was integrated at the rDNA via a transposable element [6]. (B) Tdh3 suppresses recombination at the rDNA. The rate of *URA3* marker loss at the rDNA repeats was determined by fluctuation analysis in the indicated strains. All pairwise comparisons are significant (t-test; wild type versus *Δsir2*, p = 0.012; *Δsir2* versus *Δtdh3*, p = 0.011; *Δtdh3* versus *Δsir2 Δtdh3*, p = 0.030).

Sir2 regulates recombination and RNA polymerase II transcription at the rDNA. To examine the influence of Tdh3 on silencing and recombination at the rDNA locus, we monitored the expression of a *URA3* reporter gene integrated into the rDNA [6]. Based on the pattern of growth on the FOA assay plates, which counterselect for *URA3* expression, loss of Tdh3 leads to a decrease in rDNA silencing and/or increased loss of the *URA3* marker (Figure 2A). To determine if Tdh3 affects recombination at the rDNA we used fluctuation analysis to measure the loss of the *URA3* marker from the rDNA repeats (Figure 2B). In agreement with prior studies we find that deletion of Sir2 increases the rate of loss of the rDNA marker [6]. We also observe a significant increase in recombination in strains lacking Tdh3. Loss of Sir2 in a *Δtdh3* strain does not cause an additive increase in the recombination rate, suggesting that Sir2 and Tdh3 act in a common pathway to suppress rDNA recombination.

### Tdh3 catalytic activity does not correlate with silencing

Silencing may be influenced by flux through the glycolytic pathway, controlled in part by Tdh3 in yeast. To examine the relationship between Tdh3's enzymatic activity and its effect on silencing we assessed the effects of mutations in the *TDH3* gene. We replaced the endogenous *TDH3* gene with alleles predicted to code for proteins that reduce Tdh3's catalytic activity (C150G) [21] and/or to alter its multimeric state (T227A, T227K) [22]. These Tdh3 proteins were expressed at similar levels to wild type (not shown).

**Figure 3:**
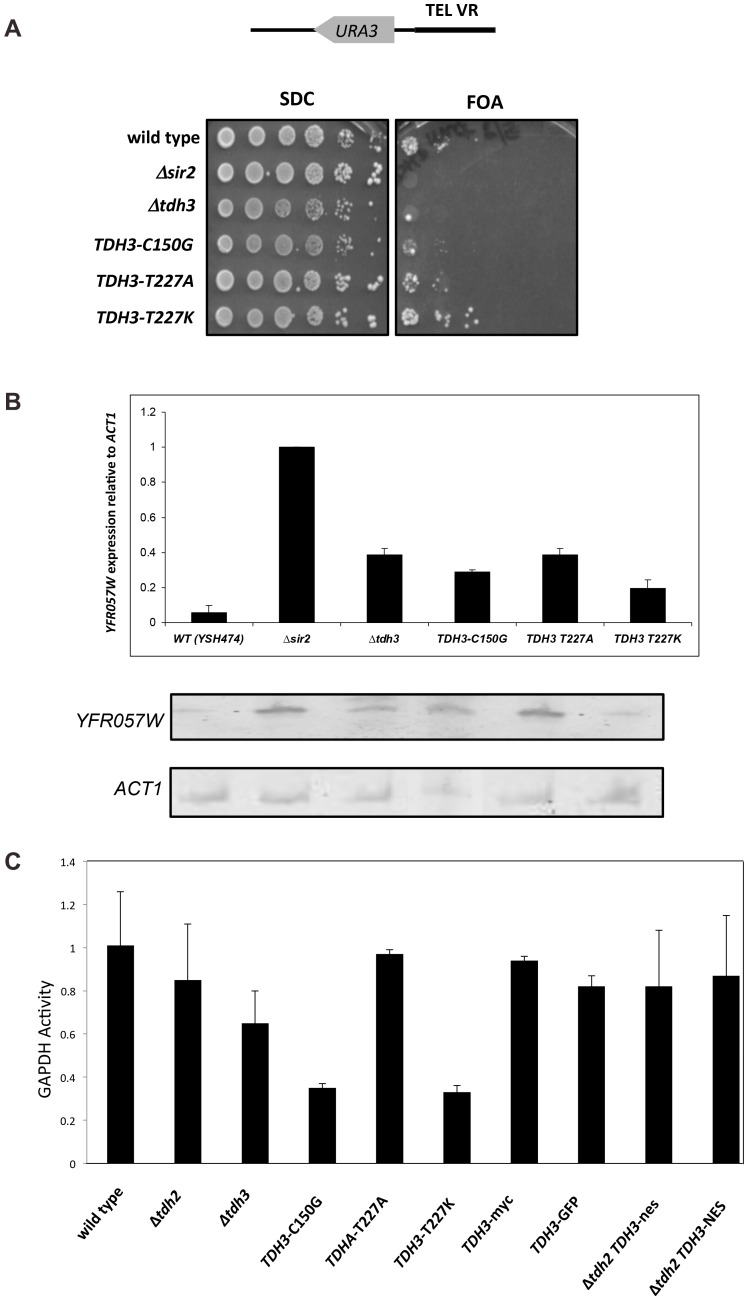
Separation of silencing and GAPDH activity in *TDH3* alleles. (A) *TDH3* mutants influence transcriptional silencing at yeast telomeres. For each allele the wild-type amino acid and position is noted, followed by the amino acid replacing it in the mutated allele. A phenotypic assay measuring silencing of a *URA3* reporter gene was conducted as described in the Figure 1 legend. (B) mRNA levels of *YFR057W*, a naturally occurring telomere proximal gene, were determined as described in the Figure 1 legend. (C) GAPDH levels of strains bearing *TDH3* alleles. Levels of glyceraldehyde phosphate dehydrogenase activity were measured in extracts made from the indicated strains, as previously described [64].

We then measured the effects of these mutants on cellular GAPDH activity and on silencing at the telomere (Figure 3). We found that GAPDH activity in the strains does not correlate with silencing efficiency. While the C150G amino acid substitution showed diminished GAPDH activity and also exhibited a decrease in silencing similar to cells lacking Tdh3, the T227A change caused a silencing defect with no change in GAPDH activity. Finally, the T227K strain exhibited no change in silencing in the phenotypic assay (Figure 3A), and only a slight loss of silencing as assessed by mRNA levels of a telomere proximal gene (Figure 3B), despite a significant drop in GAPDH activity. Thus, Tdh3 likely contributes to silencing in a manner that is at least partly independent of its role in glycolysis. Interestingly, we observed that expression of specific Tdh3 mutants (e.g., C150G and T227K) caused GAPDH activity to drop below levels seen in the *Δtdh3* null strain (Figure 3C). The active form of the GAPDH enzyme is a tetramer of GAPDH monomers. The existence of mixed Tdh2/Tdh3 tetramers has been suggested [13]; we speculate that expression of specific Tdh3 alleles could decrease overall GAPDH activity by recruiting Tdh2 into inactive complexes.

### Nuclear localization of Tdh3 is required to maintain transcriptional silencing

**Figure 4:**
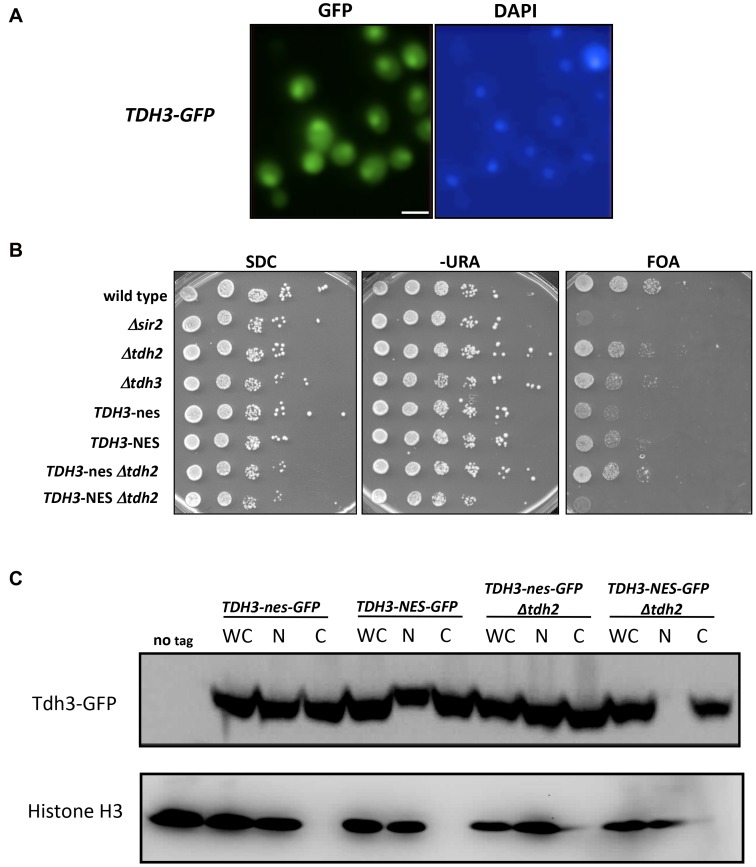
Nuclear localization of Tdh3 influences transcriptional silencing at the telomere. (A) Tdh3 is localized to both the cytoplasm and nucleus. Cells expressing Tdh3-GFP from the native *TDH3* locus were visualized by fluorescence microscopy. Size bar: 5 µm. (B) Addition of a nuclear export sequence to Tdh3 reduces silencing at the telomere. The indicated alleles of *TDH3* were introduced at its endogenous loci in a strain bearing the *URA3* gene adjacent to a telomere. NES denotes a functional nuclear export sequence; nes denotes a non-functional sequence that differs by two amino acid substitutions [27]. Expression of *URA3* was assessed by plating serial dilutions of these strains on the indicated media. (C) Localization of Tdh3-NES-GFP and Tdh3-nes-GFP was examined by cellular fractionation and immunoblotting. Fractions of the indicated strains were probed using an antibody to GFP. Detection of histone H3 was used to monitor the success of fractionation. Fractions included whole cell (WC), nuclear (N), and cytoplasmic (C). Localization of Tdh3-GFP was also examined by fluorescent microscopy (Supplemental Figure S1A). Addition of the GFP tag to Tdh3 in NES/nes strains did not alter their silencing phenotypes (Supplementary Figure S1B).

We find that yeast GAPDH, which participates in glycolysis in the cytoplasm, also influences silencing and recombination in the nucleus. This influence could be indirect, reflecting in some way the key role these enzymes play in basic cell metabolism. However, GAPDH enzymes in other organisms have been shown to exist in the nucleus and execute functions independent of their role in glycolysis [21], [23], [24]. We examined the possibility that yeast Tdh3 protein is a nuclear factor in yeast with a direct role in silencing. We first used a strain expressing a Tdh3-GFP fusion protein to determine the cellular localization of Tdh3. Monitoring GFP by fluorescence microscopy indicated that Tdh3 in present in both the nucleus and cytoplasm (Figure 4A), consistent with reports from large-scale localization efforts [25]. We observed a similar pattern performing immunofluorescence of a strain expressing a Tdh3-myc fusion protein (not shown). We next asked whether nuclear localization was important for Tdh3's function in silencing by fusing a nuclear export sequence (NES) to the C-terminus of Tdh3. We used a 12 amino acid NES derived from the HIV Rev1 protein, previously shown to be functional in yeast [26]. As a control we fused Tdh3 to a non-functional sequence (“nes”) that differs at two key amino acid positions [27]. We created strains expressing this allele in otherwise wild-type strains, and in strains lacking the *TDH2* gene. In both *TDH2* and *Δtdh2* strains, addition of NES or nes sequences to Tdh3 did not lead to noticeable changes in cell growth, nor did they significantly alter overall GAPDH levels in the cell (Figure 3C). We did not observe a difference in silencing between the NES- and nes-tagged strains in an otherwise wild-type strain, but observed a significant, specific loss of silencing when the NES sequence is fused to Tdh3 in a strain lacking the Tdh2 protein (Figure 4B). We used GFP-tagged versions of these strains to show that addition of the NES sequence in *Δtdh2* strains, but not the nes sequence, led to a redistribution of Tdh3 protein (Figure 4C). We did not observe a significant change in the distribution of Sir2 in these strains (Supplementary Figure S1C). Overall these experiments suggest that Tdh3 is present in the nucleus, and that nuclear localization is important for its role in silencing. They also suggest that Tdh2 affects Tdh3's localization in the cell. Finally, we note that the *Δtdh2 TDH3-NES* strain that exhibits defective silencing has normal levels of GAPDH activity (Figure 3C), further suggesting that Tdh3's contribution to silencing is independent of its ability to perform catalysis.

### Tdh3 and Sir2 physically interact *in vivo*

**Figure 5:**
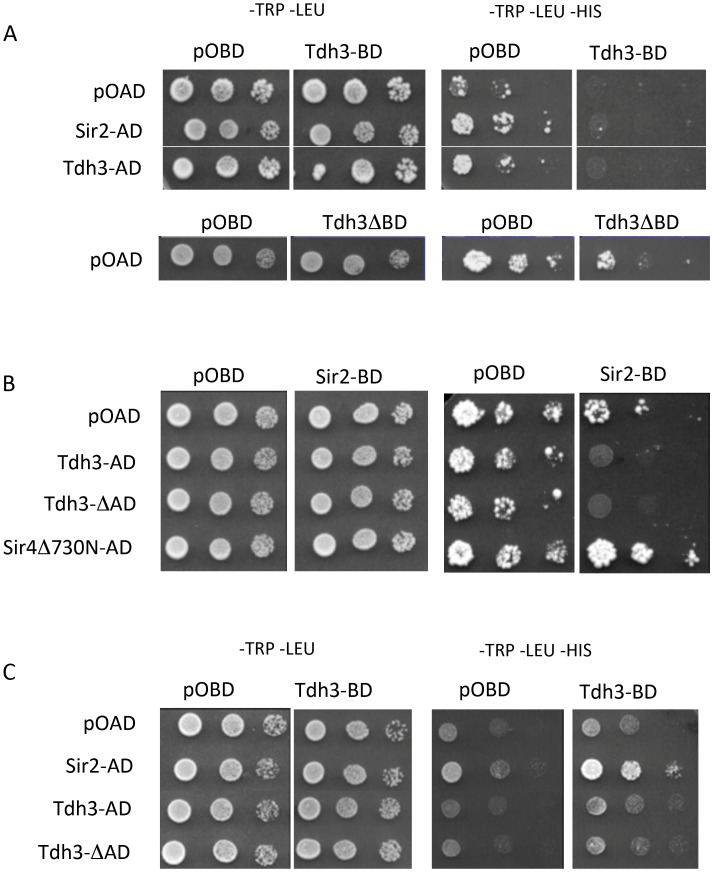
Physical and functional interaction between Tdh3 and Sir2 in a two-hybrid assay. (A) *TDH3* fused to the DNA-binding domain results in the repression of the *HIS3* reporter gene. Two-hybrid assays were performed as previously described [69], [70] using the complete Sir2 and Tdh3 open reading frames. Rows are labeled with the activation-domain fusions used; pOAD is the vector control. Each column lists the binding-domain fusion used; pOBD is the vector control. Tdh3ΔBD indicates strains that overexpress *TDH3* from the pOBD vector lacking the Gal4 binding domain. (B) Elevated Tdh3 increases Sir2-dependent repression of a reporter gene. Labels are as described in (A). Sir4Δ730N-AD was included as a positive control for Sir2 interaction. (C) Tdh3 and Sir2 interact in vivo. The activation domain and binding domain fusions from (A) and (B) were assessed in a strain lacking the *SIR2, SIR3, and SIR4* genes (YSH625).

To examine the possibility that Sir2 and Tdh3 physically interact, we fused Sir2 and Tdh3 to the DNA binding domain (BD) or transcriptional activation domain (AD) of the Gal4 protein and expressed the fusion proteins in a strain bearing Gal4 binding sites in the *HIS3* promoter. In initial experiments we failed to see evidence of a Tdh3-Sir2 interaction, but we noticed that the Tdh3-BD protein significantly repressed basal expression of the *HIS3* reporter gene (Figure 5A). To determine if the repression mediated by Tdh3 required DNA binding, we expressed Tdh3 lacking the DNA binding domain. Basal *HIS3* expression is restored in these conditions, suggesting that tethering Tdh3 caused transcriptional repression (Figure 5A, lower panel).

When Sir2 was tethered to the *HIS3* promoter via fusion with the Gal4 DNA binding domain and Tdh3 was expressed as an activation domain fusion, we again failed to observe evidence of a Tdh3-Sir2 interaction. In these experiments tethered Sir2 alone does not repress the reporter, consistent with previous reports. However, expression of Tdh3 in conjunction with tethered Sir2 caused repression of *HIS3*. Thus, increased Tdh3 in the cell appears to increase an intrinsic ability of Sir2 to mediate tethered silencing (Figure 5B).

The presence of a positive interaction in two hybrid assays can be masked by the ability of the query proteins to repress transcription of the reporter gene. To reduce this possibility we repeated the two-hybrid assay in a strain lacking the endogenous *SIR2*, *SIR3*, and *SIR4* genes [28]. In contrast to the Sir^+^ strain, expression of the Tdh3-BD protein in the *sir2 sir3 sir4* mutant strain does not alter basal expression of *HIS3* (Figure 5C). Finally, when the Tdh3-BD fusion is expressed along with Sir2-AD, we observed increased growth on –HIS media, indicating an interaction between the two proteins (Figure 5C).

**Figure 6:**
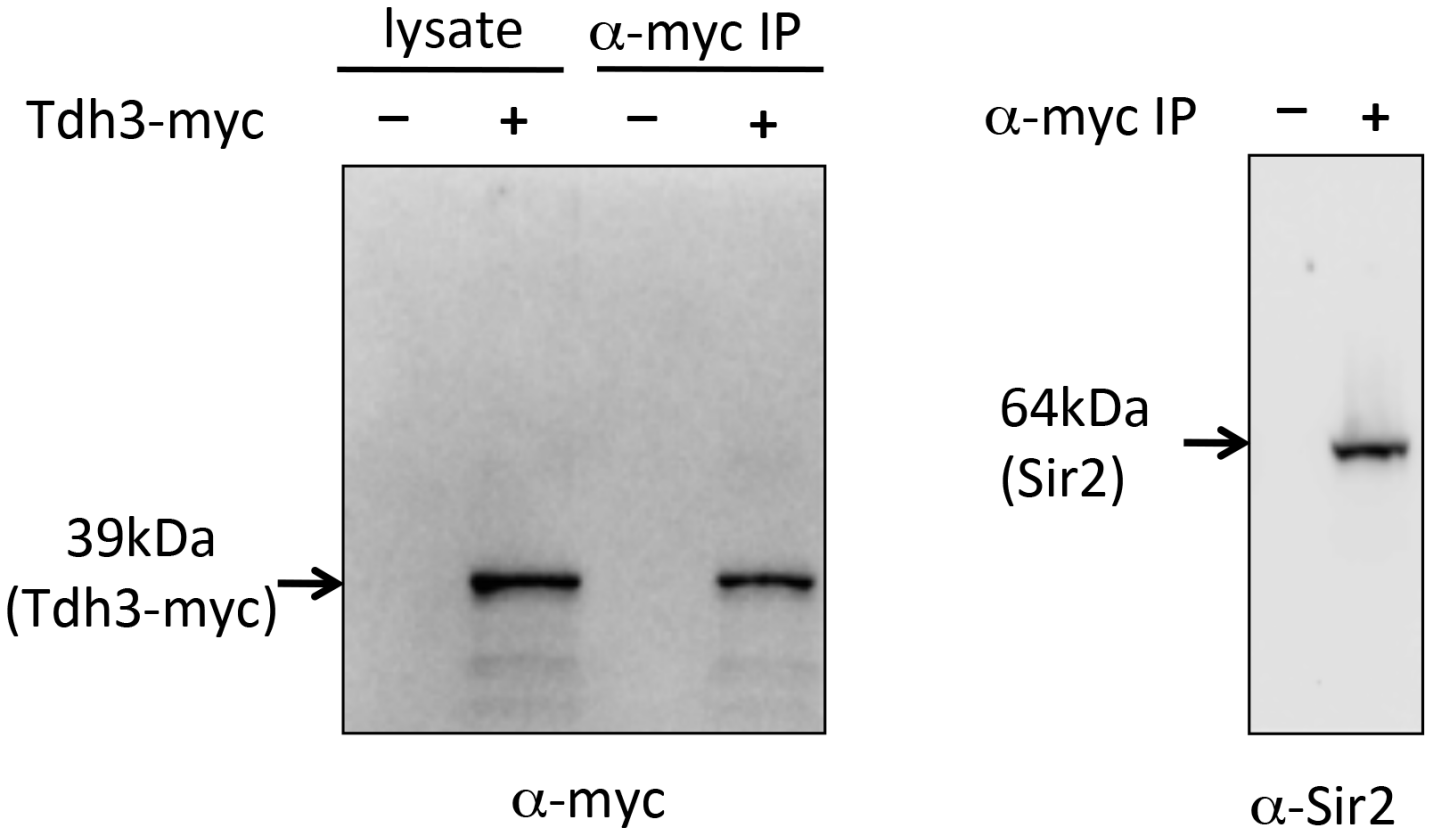
Co-immunoprecipitation of Tdh3 and Sir2. A Tdh3-myc fusion protein was immunoprecipitated from yeast cell lysates. The panel on the left shows a western blot probed with anti-myc antibody. Lanes include crude lysate and immunoprecipitated material (IP). Control lysates were made from strains lacking the myc tag on Tdh3. The right panel shows a western blot of the same immunoprecipitated material, probed with an antibody to Sir2. This antibody specifically recognizes Sir2 (Figure 1D).

As an independent approach to assess a possible Tdh3-Sir2 interaction we carried out a co-immunoprecipitation experiment. For this experiment we made a strain expressing a Tdh3-myc fusion protein, transribed from the endogenous *TDH3* locus. Extracts were made from this strain, and from a control strain lacking the myc tag. Tdh3-myc and associated proteins were separated from crude cellular extracts using antibodies to myc conjugated to agarose beads. Western blotting demonstrated that Tdh3-myc was specifically detected in the cell lysate and in immunopurified fractions (Figure 6, left panel). We then ran the immunopurified material and conducted a western blot using an antibody to Sir2. The right panel of Figure 6 demonstrates that we readily detected Sir2 in immunoprecipitations from strains with tagged Tdh3, but not from control lysates treated identically but from strains lacking the myc tag on Tdh3. Our results are consistent with the results of a systematic mass spectrometry study that also suggested the existence of a complex containing Tdh3 and Sir2 [29]. Interestingly, we have failed to observe a Sir2-Tdh2 interaction under the same conditions (R. Ryznar, unpublished). Thus, our two hybrid and co-immunoprecipitation results indicate that Tdh3 specifically associates with Sir2 in yeast.

### Tdh3 is a chromatin-associated protein that regulates Sir2 association with DNA

**Figure 7:**
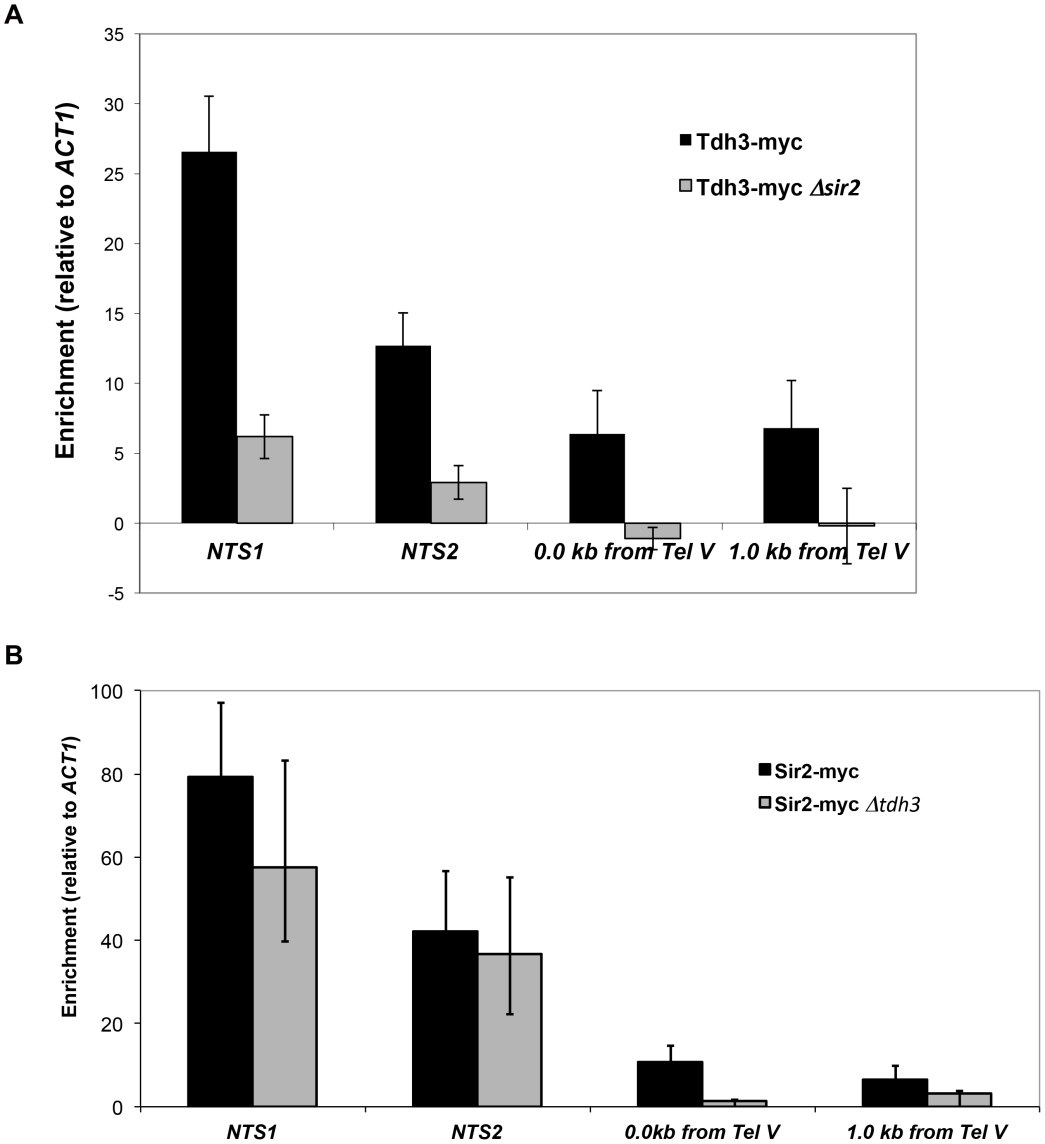
Tdh3 is present at Sir2-silenced loci. (A) The association of a Tdh3-myc fusion protein at Sir2-silenced loci was measured using chromatin immunoprecipitation, as described in Materials and Methods. Enrichment at two positions adjacent to telomere V (immediately adjacent to telomere repeats and 1 kb from telomere repeats) and two positions within the rDNA locus (NTS1 and NTS2; see Figure 2A) were assessed. Enrichment values were normalized to input DNA, and then expressed as a ratio to the normalized *ACT1* enrichment. Supplementary Figure S2 shows the same data expressed as % of input DNA precipitated. Addition of the myc tag to Tdh3 does not affect transcriptional silencing (Supplementary Figure S2A). (B) The association of a Sir2-myc fusion protein at the rDNA repeats and telomeres was assessed in *TDH3* and *Δtdh3* strains.

To examine the possibility that Tdh3 is a chromatin protein, we conducted chromatin immunoprecipitation (ChIP) experiments using a strain expressing a Tdh3-myc fusion protein. Using probes to the non-transcribed spacer (NTS) regions of the rDNA and a telomere proximal sequence, we found that Tdh3 is specifically associated with these regions of the chromosome (Figure 7A). We next determined whether Tdh3 association with chromatin depended on the presence of Sir2 by repeating these measurements in a *Δsir2* strain. We find that association of Tdh3 is eliminated at the telomere and strongly reduced at the rDNA in strains lacking Sir2. We then conducted the reciprocal experiment, examining the association of Sir2 with the rDNA and telomeres in strains lacking the *TDH3* gene (Figure 7B). In these experiments we observe a reduction of Sir2 association with telomeres, but don't observe a significant decrease at the rDNA (Figure 7B). Therefore, Tdh3 is a chromatin protein that regulates the ability of Sir2 to associate with some silent loci.

### Tdh3 regulates nuclear NAD^+^ levels

**Figure 8:**
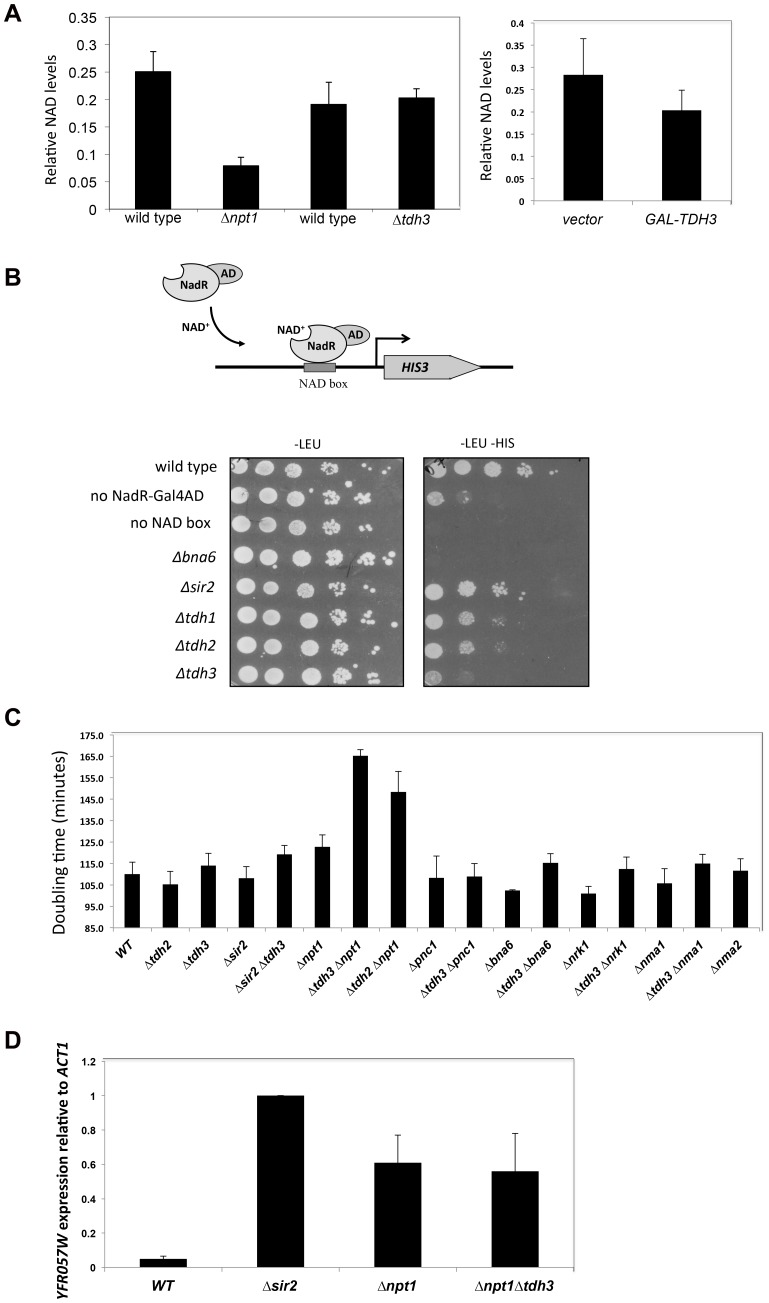
Tdh3 affects nuclear NAD^+^ levels in yeast. (A) *TDH3* deletion or overexpression does not affect overall cellular NAD^+^ levels. Left panel: relative NAD^+^ levels are shown for strains lacking the *TDH3* or *NPT1* genes, and their matched wild-type strains. Right panel: relative NAD^+^ levels are shown in a strain overexpressing the *TDH3* gene and in a vector control strain. (B) Tdh3 maintains nuclear NAD^+^ levels. Nuclear NAD^+^ was measured using an NAD**^+^**-sensitive transcriptional reporter gene [31]. Strains expressed the NAD**^+^**-dependent transcriptional activator from a *LEU2*-marked plasmid. Control strains lacked the binding site for the transcriptional activator (no NAD box) or lacked the activator (no NadR-Gal4AD). Serial dilutions of the listed strains were plated on the indicated media. Levels of the NadR-Gal4AD protein were similar in wild type and *Δtdh3* cells (Supplementary Figure S3). (C) Tdh3 and Npt1 have a redundant role in promoting cell growth. Doubling times of the indicated single and double mutant strains is shown. (D) Silencing at the telomere in *Δnpt1 Δtdh3* strains. Expression of the native telomere gene *YFR057W*, was examined by quantitative RT-PCR in the indicated strains.

Sir2 requires NAD for its enzymatic activity, and mutations in genes that affect NAD**^+^** biosynthesis are known to influence silencing [10], [11]. GAPDH enzymes bind NAD**^+^** to catalyze a key step in glycolysis in which NAD**^+^** is reduced to NADH. Tdh3 could be affecting Sir2 activity by influencing NAD**^+^** levels in the cell. To examine whether Tdh3 gene dosage affects overall cellular NAD**^+^** levels, we measured cellular NAD**^+^** in strains lacking or overexpressing Tdh3 (Figure 8A). As a control for these experiments, we also determined the relative levels of NAD**^+^** in a strain lacking the *NPT1* gene, a mutation reported to decrease cellular NAD**^+^** [10]. We readily detected a decrease in NAD**^+^** levels in the *Δnpt1* strain relative to its wild-type control, but failed to detect a significant change in strains lacking Tdh3 (Figure 8A, left panel) or overexpressing Tdh3 (Figure 8A, right panel).

Several studies suggest that NAD**^+^** concentration may vary depending on cellular compartment [30]. To examine the possibility that Tdh3 specifically affects levels of NAD**^+^** within the nucleus, we used the NAD**^+^**-sensitive transcriptional reporter described by Anderson et al [31]. In this strain the bacterial NadR protein is fused to the Gal4 activation domain, while binding sites for NadR are present in the *HIS3* gene promoter. NadR's binding to DNA depends on the presence of NAD**^+^**; thus, transcription of *HIS3* is tightly linked to nuclear NAD**^+^** availability (Figure 8B). We used this assay to measure the effects of eliminating Tdh1, Tdh2, Tdh3, Sir2, or Bna6, an enzyme known to influence nuclear NAD**^+^** levels [31]. We observed a significant and specific decrease in reporter expression in a strain lacking the *TDH3* gene, suggesting that the Tdh3 protein helps maintain normal nuclear NAD**^+^** levels (Figure 8B). *HIS3* expression is also reduced in this assay in *Δtdh2 TDH3-NES* and *Δtdh2 TDH3-nes* strains (Supplementary Figure S4).

Proteins contributing to common pathways in the cell can often be identified by defining synthetic phenotypes caused by combining mutations in the genes for these proteins [32]. To further examine Tdh3's possible role in maintaining cellular NAD**^+^** levels we created strains combining *TDH3* deletions with the loss of genes involved in the synthesis of NAD**^+^**, and then compared the doubling times of strains containing the single and double mutations. Interestingly, we observed a significant slow-growth phenotype in a strain lacking both the *TDH3* and *NPT1* genes (Figure 8C), consistent with an observation made in a large-scale assay [33]. We detected a similar growth defect in a *Δtdh2 Δnpt1* strain (Figure 8C). Npt1 is largely found in the nucleus [10], [34], where it participates in the salvage pathway of NAD^+^ synthesis. Consistent with prior studies [10], [35], we observed that *Δnpt1* strains exhibited silencing defects; we also found that cells lacking both *TDH3* and *NPT1* have silencing defects similar to those seen in *Δnpt1* or *Δtdh3* strains (Figures 1C and 8D).

## Discussion

### Tdh3 is a chromatin protein that promotes Sir2-dependent silencing

GAPDH is a well-described “moonlighting” protein, shown to have diverse functions independent of its role in glycolysis [23], [36]. These functions may include a conserved interaction with Sir2 family members, as GAPDH enzymes have been shown to interact with sirtuins in other organisms. In *Drosophila*, a large-scale two-hybrid interaction study indicated an interaction between GAPDH and dSir2 [37], while in human cells the nitrosylated form of GAPDH was shown to bind to SIRT1, the closest human homologue to yeast Sir2, and lead to SIRT1 nitrosylation [38]. GAPDH translocation to the nucleus promotes apoptosis in mammalian cells; an independent study found that SIRT1 depletion led to nuclear translocation of GAPDH in the absence of apoptotic stress [39]. Sir2-GAPDH links have also been observed in yeast cells. A recent report found that Sir2 and the Sir2 homolog Hst1 associate with the open reading frame of *TDH3* and several other glycolysis genes, and may mediate repression of these genes following the diauxic shift [40]. Overexpressing Sir2 in GAPDH-deficient yeast cells caused elevated plasmid recombination [41], prompting a proposal that GAPDH enzymes influence Sir2 activity, possibly by affecting availability of its cofactor, NAD**^+^** [41], [42].

We previously identified Tdh3 in a screen for possible regulators or substrates of Sir2 [12]. Here we report that strains lacking Tdh3 have defects in telomere position effect and rDNA silencing. We also found that Tdh3 physically interacts with Sir2, and specifically binds to both telomeres and rDNA sequences in a Sir2-dependent manner. Finally, Sir2's association with telomeres was reduced in strains lacking Tdh3. Taken together, these observations suggest that Tdh3 acts directly at the sites of Sir2 action to influence silencing. Our experiments suggest that Tdh3 promotes silencing in yeast cells independently of its role in glycolysis. First, Tdh3's silencing activity was decreased by the addition of sequences that promoted its export from the nucleus. Thus, unlike its function in glycolysis, Tdh3's role in silencing likely occurs in the nucleus. Second, our analysis of a small set of Tdh3 mutants indicated that its ability to promote silencing did not correlate with catalytic activity. Given its association with Sir2 at its chromatin targets, Tdh3 may affect silencing directly by influencing Sir2's catalytic activity or its interaction with other silencing factors. Since Tdh3 is an NAD^+^-binding protein that reduces NAD^+^ to NADH during glycolysis, we also investigated this possible link to Sir2. While we observed that overall NAD**^+^** levels are unchanged in cells lacking Tdh3, using an NAD**^+^**-sensitive reporter assay we found that Tdh3 is specifically required to maintain normal levels of NAD**^+^** in the nucleus. This result is consistent with the proposal that NAD**^+^** is non-uniformly distributed within the cell, in part due to compartmentalization of enzymes responsible for NAD**^+^** synthesis or consumption [30]. For instance, the yeast Npt1 enzyme involved in the NAD**^+^** salvage pathway in yeast is preferentially found in the nucleus [10], [34].

The effect of Tdh3 on nuclear NAD+ levels suggests that this GAPDH protein may influence Sir2-dependent silencing by affecting the level of NAD**^+^** available to Sir2. The Km for NAD**^+^** in Sir2's deacetylase reaction is approximately 30 µm [43] while the concentration of NAD**^+^** in yeast is between 1 and 2 mM [11]. However, genetic alterations in NAD**^+^** biosynthetic enzymes that cause silencing defects do not reduce NAD**^+^** concentrations below 1 mM; this suggests that most of the NAD**^+^** in the cell is not freely available, and is likely protein bound [11], [44]. Perhaps the NAD+ bound to Tdh3, one of the most abundant proteins in the cell, is specifically accessible to Sir2 within the nucleus. We observed that both the *Δtdh2 TDH3*-*NES* and *Δtdh2 TDH3*-*nes* strains exhibited nuclear NAD deficits, as assessed by the NadR reporter system, yet a silencing phenotype was specifically observed in the *TDH3-NES* strain, in which Tdh3's nuclear localization is reduced. Thus, silencing may be sensitive to the presence of NAD^+^-bound Tdh3 at silenced locations, rather than overall nuclear NAD levels. Finally, we note that the C150G amino acid substitution in Tdh3 that eliminates catalytic activity and which is defective in silencing is also predicted to be deficient in NAD^+^ binding [44], [45].

Due to its role in regulating aging in yeast and in other organisms, particularly for its proposed role in mediating the effects of calorie restriction in the aging pathway, potential links between metabolism and Sir2 function have been actively sought [2], [8], [9], [42]. The effects of calorie restriction (CR) on yeast lifespan act through Sir2-dependent and Sir2 independent mechanisms [45], [46], and it is not clear if CR influences Sir2 activity by modulating NAD**^+^** levels [45], [47], [48], [49]. We have found that Tdh3 has functions in basic cell metabolism and control of Sir2-induced transcriptional silencing. Tdh3 thus exhibits the hallmarks of a factor that could link cellular metabolism with Sir2-dependent silencing.

## Materials and Methods

### Strains and plasmids

Strains used in this study are listed in Table 1. Genes were eliminated by PCR-mediated gene deletion [50], using MX-series plasmids as templates [51]. Epitope tags were fused to the 3′ end of targeted via PCR-mediated insertion using plasmid pYM5 as template [52].

**Table 1:** Strains.

Strain	Genotype	Source
YSH503 (CCFY100)	*MAT*a W303-1A *ade2-1 ura3-1 trp1-289 leu2-3,112 his3-11,15 can1-100 hmr Δ E::TRP1 rDNA::ADE2-CAN1 TELVR::URA3*	[17]
YSH529	YSH503; *Δsir2::KAN*	[17]
YSH878	YSH503; *Δtdh1::NAT*	
YSH879	YSH503; *Δtdh2::NAT*	
YSH880	YSH503; *Δtdh3::KAN*	
YSH387 (YLS404)	*MATA hmrΔA::ADE2 ade2-1 can1-100 his3-11, 15 leu2-3, 112 trp1-1 ura3-1 GAL+hmrΔA::ADE2*	[71]
YSH1041	YSH503; *tdh3-C150G*	
YSH1093	YSH503; *tdh3-T227A*	
YSH1094	YSH503; *tdh3-T227K*	
YSH615 (DMY480)	*MATα his3 Δ200 leu2 Δ1 ura3-167 RDN::Ty1-mURA3*	
YSH614 (DMY1097)	YSH615; *Δsir2::HIS3*	
YSH883	YSH615; *Δtdh3::KAN*	
YSH882	YSH615; *Δtdh3::NAT*	
YSH913	YSH883; *Δsir2::NAT*	
YSH961	YSH474; *TDH3-(3xMyc)-HIS3*	
YSH905	YSH474; *TDH3-GFP*(S65T)–*HIS3MX*	[25]
YSH964	YSH503; *Δtdh2::NAT TDH3-nes-HYG*	
YSH965	YSH503; *Δtdh2::NAT TDH3-NES-HYG*	
YSH962	YSH503; *TDH3-nes-HYG*	
YSH963	YSH503; *TDH3-NES-HYG*	
YSH513 (YMM400)	*MATa trp1-901 leu2-3, 112 ura3-52 his3-200 gal4Δ gal80Δ LYS2::GAL1-HIS3 GAL2-ADE2 met2::GAL7-lacZ*	[69]
YSH514 (YMM401)	*MAT α trp1-901 leu2-3, 112 ura3-52 his3-200 gal4Δ gal80Δ LYS2::GAL1-HIS3 GAL2-ADE2 met2::GAL7-lacZ*	[69]
YSH625	*trp1-901 leu2-3,112 ura3-52 his3-200 gal4Δ gal 80Δ LYS2::GAL1-HIS3 GAL2-ADE2 met2::GAL7-lacZ Δsir3::nat1 Δsir4::URA3 Δsir2::HYG*	[72]
YSH474 (BY4741)	*MAT* a *his3Δ1 leu2Δ0 met15Δ0 ura3Δ0*	Open Biosystems
YSH961	YSH474; *TDH3-(3xMyc)-HIS3*	
YSH974	YSH961; *Δsir2::NAT*	
YSH621	*his3Δ200 leu2Δ0 met15Δ0 trp1Δ63 ura3Δ0 SIR2-3Xmyc-HIS3 MX*	
YSH984	YSH621; *Δtdh3::HYG*	
YSH506 (JS237)	*MAT*a *his3Δ200 leu2Δ1 met15Δ0 trp1Δ63 ura3-167 RDN1::*Ty1-*MET15*	[6]
YSH507 (JS596)	*MAT α his3Δ300 leu2Δ1 met15Δ0 trp1Δ63 ura3Δ167 RDN1::Ty1-MET15 npt1::kanMX*	[35]
YSH695	YSH474; *Δtdh3::KAN*	
YSH896 (NS464)	*leu2 his3* (4× Nad boxes)-*HIS3*	[31]
YSH897	*leu2 his3* (4× mutated Nad boxes)-*HIS3*	[31]
YSH898	YSH896; *Δbna6*	[31]
YSH901	YSH896; Δ*sir2::NAT*	
YSH902	YSH896; *Δtdh1::NAT*	
YSH903	YSH896; *Δtdh2::NAT*	
YSH904	YSH896; *Δtdh3::KAN*	

To introduce mutated alleles of the *TDH3* gene a strain was made in which *TDH3* was replaced by the pCORE construct [53]. DNA fragments containing specific point mutations in *TDH3* were made by hybrid PCR [54] and used to transplace the pCORE sequences. Alleles were confirmed by sequencing.

Nuclear export sequences were fused to the 3′ end of *TDH3* by transforming a DNA fragment with 3′ homology to the *TDH3* ORF, the nuclear export sequence, and an *hphMX4* sequence into the appropriate yeast strain. Strains lacking both *TDH3* and specific NAD**^+^** biosynthetic genes were generated by crossing *Δtdh3* strain YSH969 with selected strains from the yeast deletion collection [55]; following sporulation haploid strains were identified by selecting for histidine auxotrophs [56].

### Immunostaining and microscopy

Semisquash preparations were adapted from published protocols [57], [58] with minor modifications [59]. Immunostaining was performed using a mouse monoclonal antibody against Nsp1p (ab4641; Abcam) at a 1∶100 dilution to mark the nuclear periphery and Alexa Fluor 568–goat anti-mouse IgG (H+L) (A11004; Molecular Probes) at a 1∶200 dilution as the secondary antibody. A chicken monoclonal antibody against GFP (ab13970; Abcam) at a 1∶100 dilution was used to recognize the Tdh3-nes-GFP or Tdh3-NES-GFP fusion constructs and FITC conjugate from Jackson Immuno Research at 1∶200 dilution was used as the secondary antibody. Nuclear to cytoplasmic ratio of GFP fluorescence was determined using the arbitrary line tool of Softworx software, in conjunction with the Deltavision RT imaging system (Applied Precision) adapted to an Olympus (IX70) microscope. Image stacks at 0.2-µm spacing were acquired along the *z* axis. The line tool was used to generate GFP fluorescence histogram profiles reflecting relative fluorescence units of the nucleus as compared to the cytoplasm.

### Chromatin immunoprecipitation

ChIP was performed as previously described [60]. Yeast cell growth and chromatin preparation were performed as described [61]. Prior to the addition of antibody for precipitation, 50 µl of lysate was precleared with 7 µl of Protein A magnetic beads (New England Biolabs) by incubating at 4°C for 30–60 minutes on a Labquake tube rotator. The samples were applied to a magnet to separate the beads from the supernatant; the supernatant was transferred to a new eppendorf tube and 1 µl myc-epitope antibody (9B11; Cell Signaling Technology) was added for an overnight incubation at 4°C). 15 µl of Protein A magnetic beads were added to precipitate the chromatin. Control (mock) immunoprecipitations were conducted in an identical manner, but without the addition of antibody.

Immunoprecipitated, control, and input DNAs were analyzed by quantitative PCR analysis. Serial dilutions of the whole cell lysate (from 1∶5 to 1∶1250) and immunoprecipitates (from 1∶2 to 1∶625) were used in a standard Taq PCR to determine a linear range for the samples, using the following cycling parameters: 94°C for 4 min; 30 cycles of 94°C for 30 s, 50°C for 30 s, and 72°C for 1.5 min; and 72°C for 5 min. For control detection of *ACT1* DNA 25 cycles of PCR was used. Data was derived only from amplifications performed within the linear range. Primers flanking non-transcribed rDNA spacers NTS1 and NTS2 were used to determine enrichment at the rDNA repeats; primers located 1.0 kb and immediately adjacent to Tel V were used to determine telomeric enrichment. Primer sequences are shown in Supplementary Table S1.

PCR products were run on 5% native polyacrylamide gel electrophoresis and stained with SYBR Gold (Invitrogen). Gels were scanned on a Storm 860 phosphorimager and quantitated using ImageQuant software (Molecular Dynamics, Inc.; Sunnyvale, CA). A sequence within the *ACT1* open reading frame was used was an internal control in all experiments. Each reported value represents the average of at least three independent ChIP experiments. For the data shown in Figure 2 the signal from each mock immunoprecipitation experiment was subtracted from the value derived from the experimental immunoprecipitation; values were then normalized to the signal observed from input DNA for each individual experiment, and then expressed as a ratio to the normalized *ACT1* value from the same experiment. The data is alternatively presented in Supplementary Figure S2 as the percentage of input chromatin precipitated, in which the signal observed from mock immunoprecipitations is reported separately.

### Co-immunoprecipitation and western blotting

For western blots protein was isolated from yeast cells as described [62]. 5 µg (for *TDH3*-myc probe) or 10 µg (for *SIR2*-myc probe) of protein was loaded onto a 5% resolving gel and 10% running gel. Protein was transferred to a nitrocellulose membrane and primary antibody applied for one hour at room temperature in 5% non-fat dry milk plus 0.1%Tween TBS solution. Anti-c-Myc (clone 9E11 from Chemicon International) was used at 1∶250 dilution. Secondary antibody (goat anti-mouse from Santa Cruz Biotechnology at 1∶3000 dilution) was applied for one hour at room temperature in the same solution. Detection was performed using the ECL Western Blotting Reagents from Amersham according to the manufacturer's specifications. Chemiluminescence was measured on a Storm PhosphorImager using the blue channel at 200 micron resolution.

For co-immunoprecipitation experiments a yeast extract was made from cells as previously described [62]), except that the triton X-100 was added to the lysis buffer to 1.5%. For immunoprecipitations 40 to 100 µl of the 1∶1 suspension of the anti-Myc agarose conjugate (Sigma) was added to a microcentrifuge tube. The resin was allowed to settle by a short microfuge spin. Liquid was discarded and washed 5 times with 1 ml ice cold PBS. Yeast cell lysate was added to the settled resin. Volume was brought to at least 200 µl (60–80 µg total protein). Tubes were incubated overnight on an orbital shaker at 4°C. The resin was washed 4 times with 1 ml of PBS. After the final wash, the supernatant was aspirated and ∼10 µl was left above the beads. 20 to 50 µl of 2× SDS sample buffer was added to the tube. The tube was incubated for 10 minutes at 92°C with frequent agitation, vortexed, and then centrifuged for 5 seconds. Carefully avoiding the agarose, the supernatant was transferred to a new tube and boiled for 5 minutes. Protein concentration was determined by Bradford assay; 20–40 µg was loaded into an SDS-PAGE gel and ran at 110 volts for 1.5 hours. Detection of the c-Myc-tagged fusion protein was determined by immunoblotting, using monoclonal anti-c-Myc for cell lysate and IP at the recommended concentration. For detection of Sir2 bound to myc tagged protein, Santa Cruz sc 2020 Sir2 antibody was used at a concentration of 1∶20. Blots were scanned using a SynGene apparatus.

### GAPDH activity assay

Assays for GAPDH activity were performed as previously described [63] with the modifications described by Ralser et al. [64].

### Cell fractionation

Cell fractionation was carried out as described [65]; western blotting of cell fractions was performed using antibodies to GFP (Abcam ab13970) and histone H3 (Abcam ab17911).

### mRNA measurements

RNA was extracted using the hot acidic phenol extraction method (Ausubel et al 1993). DNAse treatment was carried out using Ambion's RNAse-free DNAse I and reaction buffer for degrading DNA (Catalog #1906). 1 µg of RNA was used in a total of 16 µl of DEPC deionized water in a microcentrifuge tube. The sample was heated for 3 minutes at 95°C and then placed on ice for 3–5 minutes. 2 µl of 10× DNAse I buffer and 2 µl DNAse I was added and the tubes incubated at 37°C for one hour. To remove the DNAse and divalent cations that can catalyze heat-mediated degradation of RNA, 5 µl of DNAse inactivation reagent was added to the tubes and the samples were mixed well. The tubes were incubated at room temperature for two minutes during which the tubes were flicked once to re-disperse the slurry. The tubes were then microcentrifuged at room temperature for two minutes to pellet the DNAse inactivation reagent. The DNAse treated RNA was transferred to a new tube and stored at −20°C.

cDNA synthesis was carried out using Ambion's Retroscript kit (Catalog #1710). To prepare cDNA from RNA, 5 µl of the DNAse treated RNA was transferred to a new microcentrifuge tube. 1 µl of oligo(dT) primer (50 µM) was added to each tube and the samples then incubated at 85°C for 3 minutes. The tubes were then placed on ice for 3 minutes and microcentrifuged briefly at 4°C. 1 µl of RT buffer, 2 µl of dNTP mix, 0.5 µl reverse transcriptase and 0.5 µl RNAse inhibitor were added to each tube. After vortexing the tubes well, the tubes were then incubated for 60–90 minutes at 42°C and then heated at 92°C for 10 minutes. The cDNA was then spun down in a microcentrifuge at 4°C to collect the condensate. 0.6 µl was used for PCR; cycling conditions were 94°C for 4 minutes and then 25 cycles (for *ACT1*) or 35 cycles (*YFR057W*) of 94°C for 30 seconds, 50°C for 30 seconds and 72°C for 90 seconds, followed by a final cycle for 72° for 5 minutes. Primer sequences are shown in Supplementary Figure S1; primer sequences useful for detecting *YFR057W* were previously described [66].

## Supporting Information

Figure S1 Tdh3-NES-GFP nuclear levels are reduced in cells lacking Tdh2. (A) Immunofluorescence microscopy was performed on cells expressing Tdh3-NES-GFP or Tdh3-nes-GFP. The ratio of nuclear to cytoplasmic Tdh3 is indicated; at least 40 cells were assessed for each strain. (B) Addition of a GFP epitope tag to the C-terminus of Tdh3 does not influence telomeric silencing phenotypes. The experiment shown in Figure 4B was repeated using GFP tagged strains. Serial dilutions of strains bearing a *URA3* reporter gene adjacent to a telomere were made on complete medium (SDC), and on media containing 5-FOA, which counterselects for *URA3* expression. (C) Addition of NES sequences to Tdh3 does not significantly alter Sir2's nuclear to cytoplasmic ratio. The extracts used for the experiment shown in Figure 4C were probed with an antibody to Sir2. The ratio of nuclear to cytoplasmic Tdh3 is indicated, based on the signal from Sir2 immunoblots. A representative blot is shown.
(PDF)

Figure S2 Tdh3 binds to telomeres and rDNA in a Sir2-dependent manner. (A) Cells expressing Tdh3-myc exhibit normal silencing. Expression of the native telomere-proximal gene *YFR057W* was examined by quantitative RT-PCR in matched strains expressing endogenous Tdh3 or Tdh3-myc. A strain lacking Sir2 was used as an unsilenced control. (B) The chromatin immunoprecipitation data presented in Figure 7 is shown, indicating the % of input DNA that was recovered for each locus. “IP” refers to the signal achieved in ChIP experiments performed with an antibody to the myc tag; “mock” indicates the signal seen in control immunoprecipitations with no antibody. Tdh3-myc association at two positions adjacent to telomere V and two positions within the rDNA repeats (NTS1 and NTS2) were assessed in *SIR2* and *Δsir2* strains. Enrichment of a sequence within the *ACT1* open reading frame was used as a negative control. (C) The association of a Sir2-myc fusion protein at the rDNA repeats, telomere VR, and the *ACT1* gene was assessed in *TDH3* and *Δtdh3* strains. (D) Representative experiments conducted to generate the data shown in Figures 7, S2B, and S2C are shown. Strains queried are listed on the left. Gels depict the signal after PCR from input chromatin, chromatin immunoprecipitated with an antibody to the myc tag (“+AB”) and from mock immunoprecipitations in which no antibody was used (“−AB”). (E) Representative control experiments to ensure linearity of the qPCR used for ChIP experiments are shown. PCR was performed as described in Materials and Methods using primers to the indicated loci. PCR was conducted on a dilution series of input chromatin DNA; a range of the dilution series is labeled in each panel.
(PDF)

Figure S3 Tdh3 does not influence levels of NadR-AD. To determine relative levels of the NadR-Gal4AD fusion protein, western blots were performed using an antibody to the Gal4 activation domain on cell lysates from wild type and *Δtdh3* strains. Protein from a strain that does not express NadR-Gal4AD was loaded in the “vector” lane. Tubulin was detected in the same protein samples to provide a loading control.
(PDF)

Figure S4 Addition of NES or nes sequences to Tdh3 in strains lacking Tdh2 results in a decrease of nuclear NAD+ levels. The nuclear NAD**^+^** assay described for the experiment shown in Figure 8B was performed on the indicated strains. NES denotes a functional nuclear export sequence; nes denotes a non-functional sequence that differs by two amino acid substitutions [27]. Nuclear NAD^+^ was measured using an NAD**^+^**-sensitive transcriptional reporter gene [31]. Strains expressed the NAD**^+^**-dependent transcriptional activator from a *LEU2*-marked plasmid. Control strains lacked the activator (no NadR-Gal4AD). Serial dilutions of the listed strains were plated on the indicated media. The observation that the *Δtdh2 TDH3*-nes strain manifests a stronger phenotype in this assay than the comparable *Δtdh2 TDH3* strain suggests that the nes sequences affect Tdh3 function or location, perhaps in a manner that is sensitized by the absence of Tdh2.
(PDF)

Table S1 Sequences of primers used for mRNA measurements and chromatin immunoprecipitations are shown in Supplementary Table 1.
(PDF)
