# Supplementary figures and images for: Yeast Tdh3 (Glyceraldehyde 3-Phosphate Dehydrogenase) Is a Sir2-Interacting Factor That Regulates Transcriptional Silencing and rDNA Recombination
Source: PLoS Genet. 2013 Oct 17;9(10):e1003871. doi: 10.1371/journal.pgen.1003871 (PMC3798266; doi:10.1371/journal.pgen.1003871)

Supplementary Figure 1A

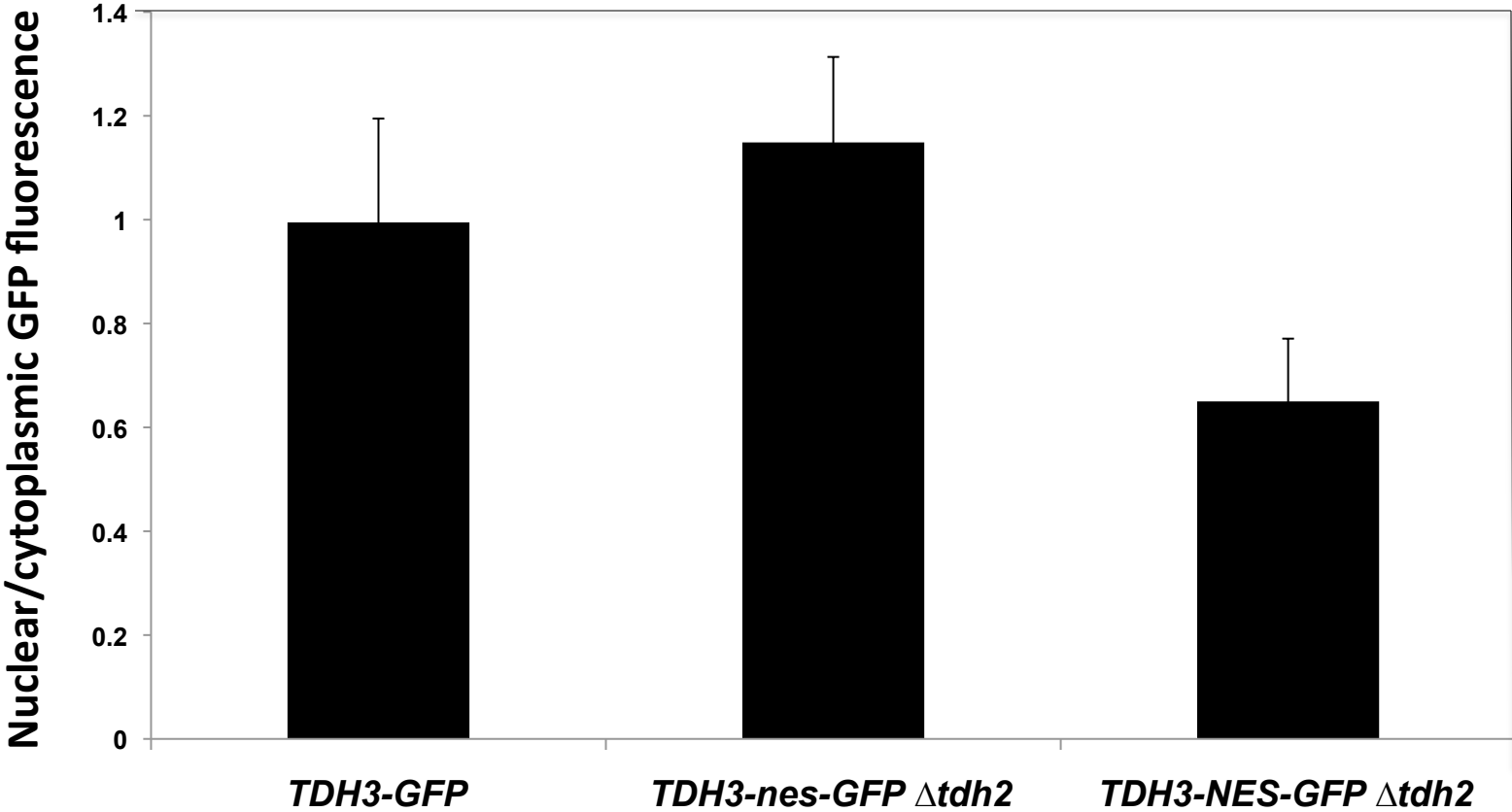

Supplementary Figure 1B

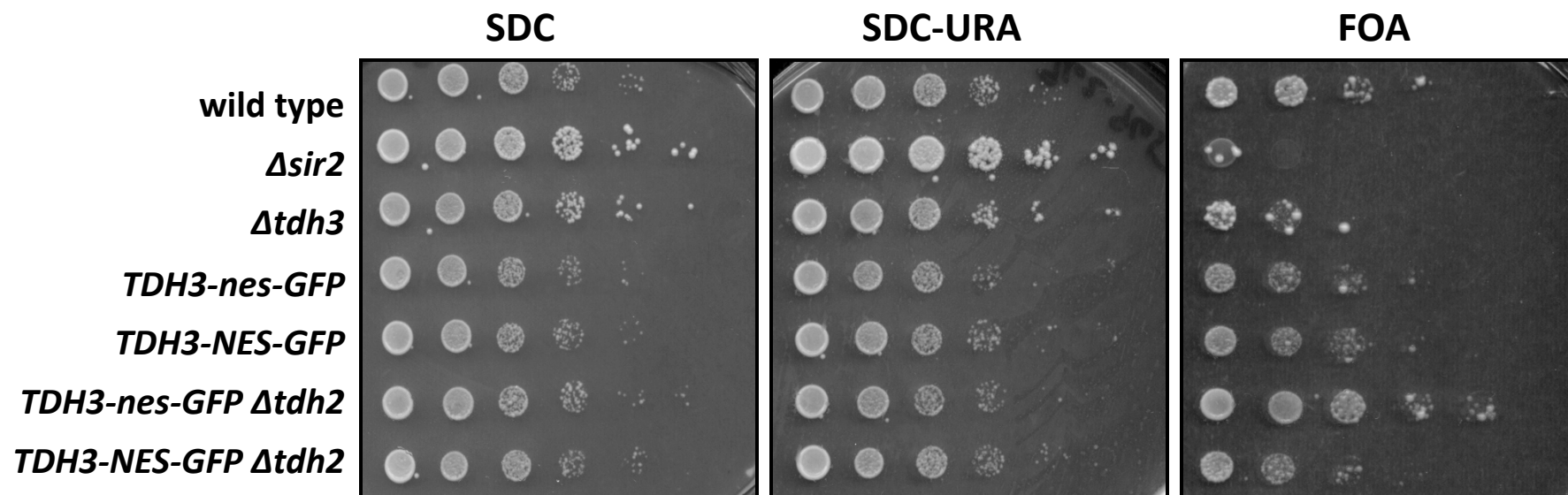

Supplementary Figure 1C

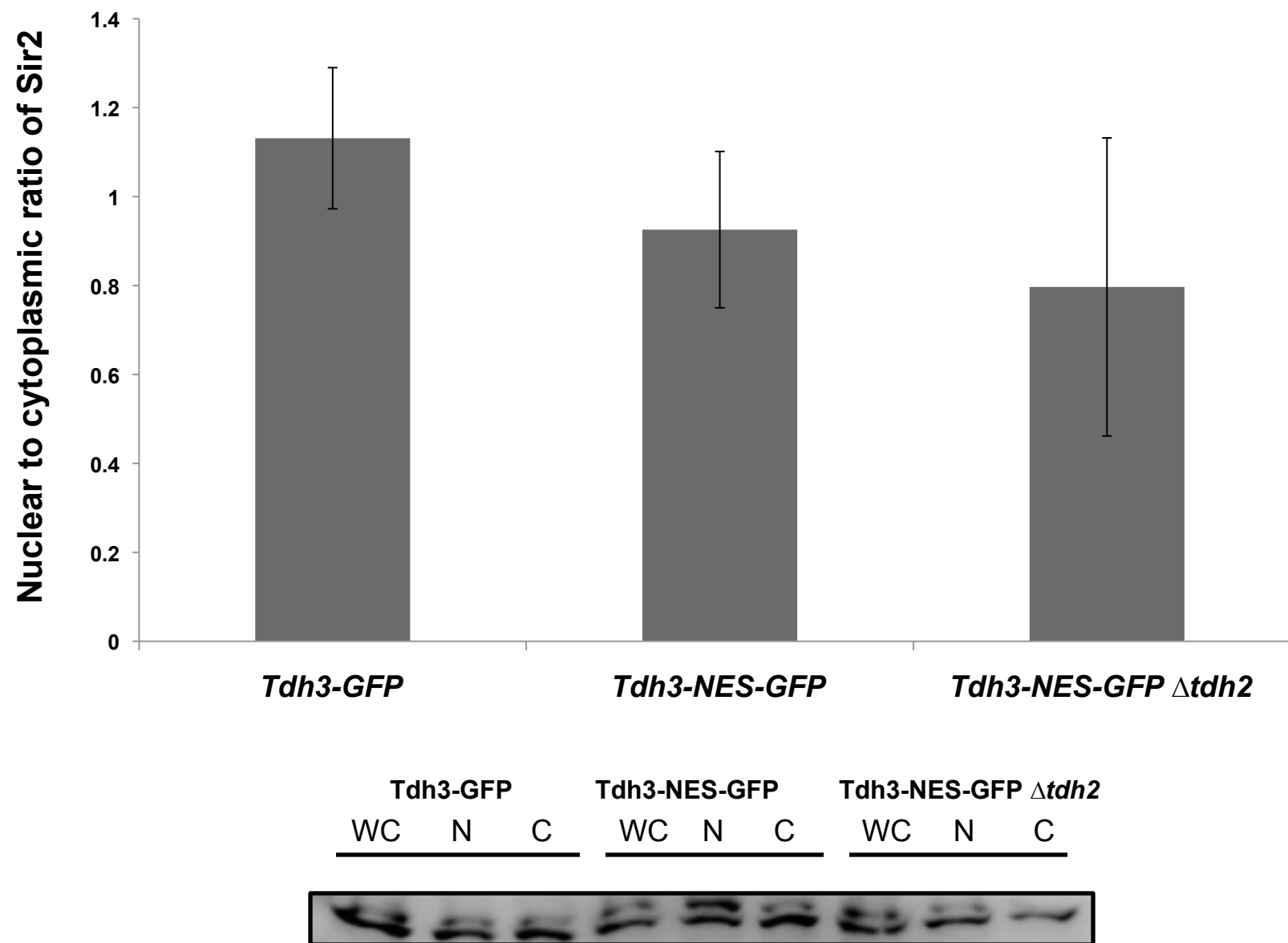

Supplement: Figure S1 — Tdh3-NES-GFP nuclear levels are reduced in cells lacking Tdh2. (A) Immunofluorescence microscopy was performed on cells expressing Tdh3-NES-GFP or Tdh3-nes-GFP. The ratio of nuclear to cytoplasmic Tdh3 is indicated; at least 40 cells were assessed for each strain. (B) Addition of a GFP epitope tag to the C-terminus of Tdh3 does not influence telomeric silencing phenotypes. The experiment shown in Figure 4B was repeated using GFP tagged strains. Serial dilutions of strains bearing a URA3 reporter gene adjacent to a telomere were made on complete medium (SDC), and on media containing 5-FOA, which counterselects for URA3 expression. (C) Addition of NES sequences to Tdh3 does not significantly alter Sir2's nuclear to cytoplasmic ratio. The extracts used for the experiment shown in Figure 4C were probed with an antibody to Sir2. The ratio of nuclear to cytoplasmic Tdh3 is indicated, based on the signal from Sir2 immunoblots. A representative blot is shown. (PDF) [file pgen.1003871.s001.pdf]

Supplementary Figure 3

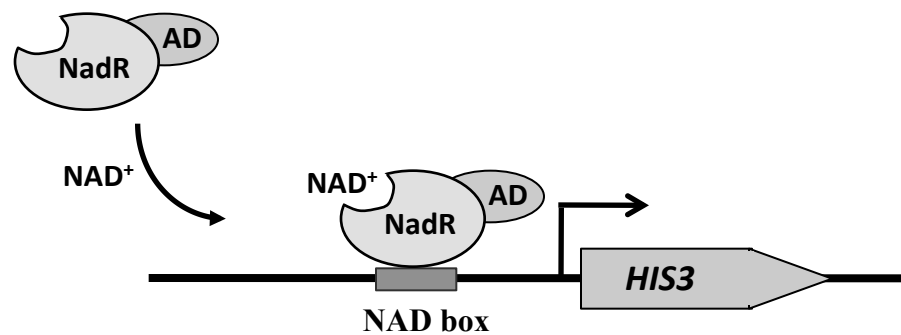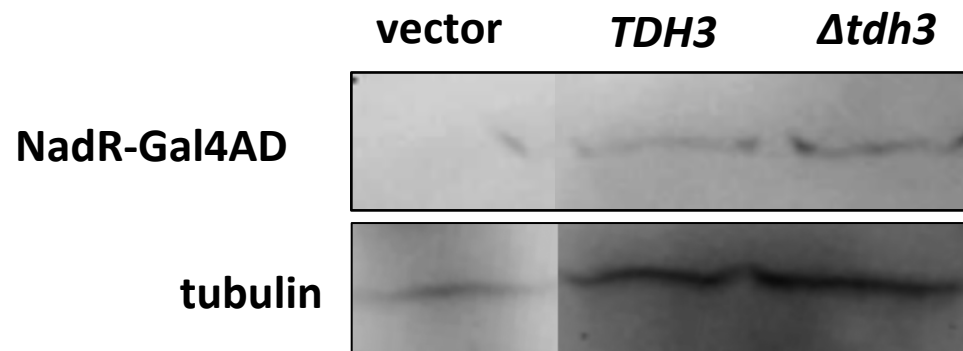

Supplement: Figure S3 — Tdh3 does not influence levels of NadR-AD. To determine relative levels of the NadR-Gal4AD fusion protein, western blots were performed using an antibody to the Gal4 activation domain on cell lysates from wild type and Δtdh3 strains. Protein from a strain that does not express NadR-Gal4AD was loaded in the “vector” lane. Tubulin was detected in the same protein samples to provide a loading control. (PDF) [file pgen.1003871.s003.pdf]

Supplementary Figure 4

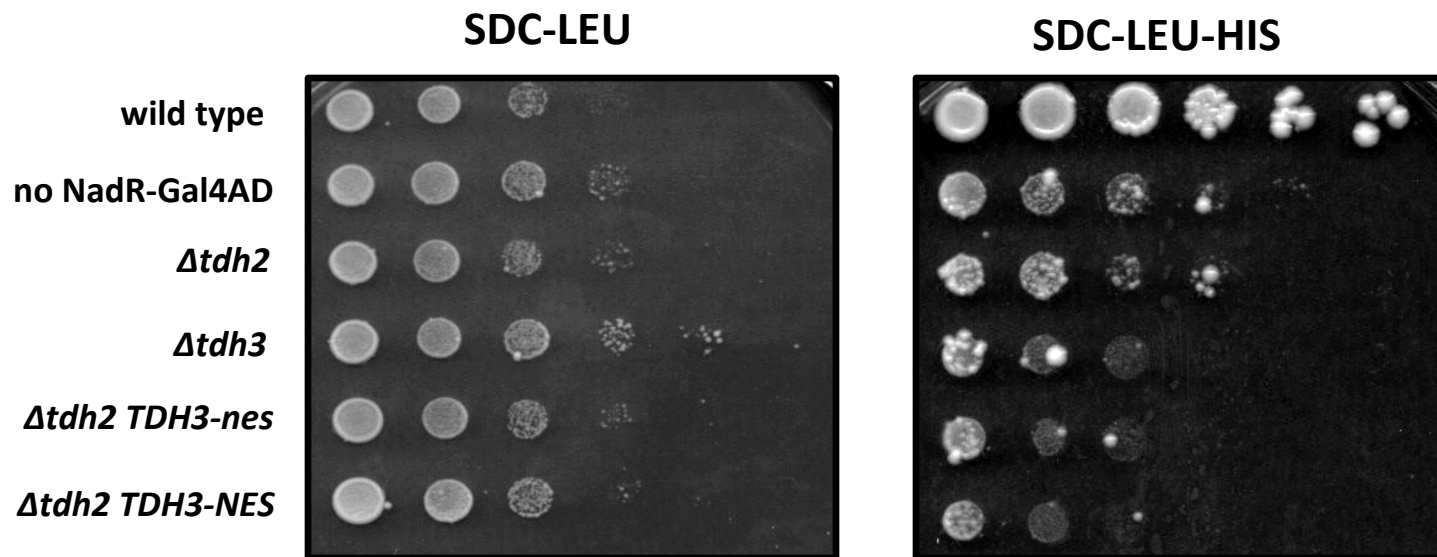

Supplement: Figure S4 — Addition of NES or nes sequences to Tdh3 in strains lacking Tdh2 results in a decrease of nuclear NAD+ levels. The nuclear NAD+ assay described for the experiment shown in Figure 8B was performed on the indicated strains. NES denotes a functional nuclear export sequence; nes denotes a non-functional sequence that differs by two amino acid substitutions [27]. Nuclear NAD+ was measured using an NAD+-sensitive transcriptional reporter gene [31]. Strains expressed the NAD+-dependent transcriptional activator from a LEU2-marked plasmid. Control strains lacked the activator (no NadR-Gal4AD). Serial dilutions of the listed strains were plated on the indicated media. The observation that the Δtdh2 TDH3-nes strain manifests a stronger phenotype in this assay than the comparable Δtdh2 TDH3 strain suggests that the nes sequences affect Tdh3 function or location, perhaps in a manner that is sensitized by the absence of Tdh2. (PDF) [file pgen.1003871.s004.pdf]
