# Supplementary material for: Yeast Tdh3 (Glyceraldehyde 3-Phosphate Dehydrogenase) Is a Sir2-Interacting Factor That Regulates Transcriptional Silencing and rDNA Recombination
Source: PLoS Genet. 2013 Oct 17;9(10):e1003871. doi: 10.1371/journal.pgen.1003871 (PMC3798266; doi:10.1371/journal.pgen.1003871)

Supplementary Figure 2A

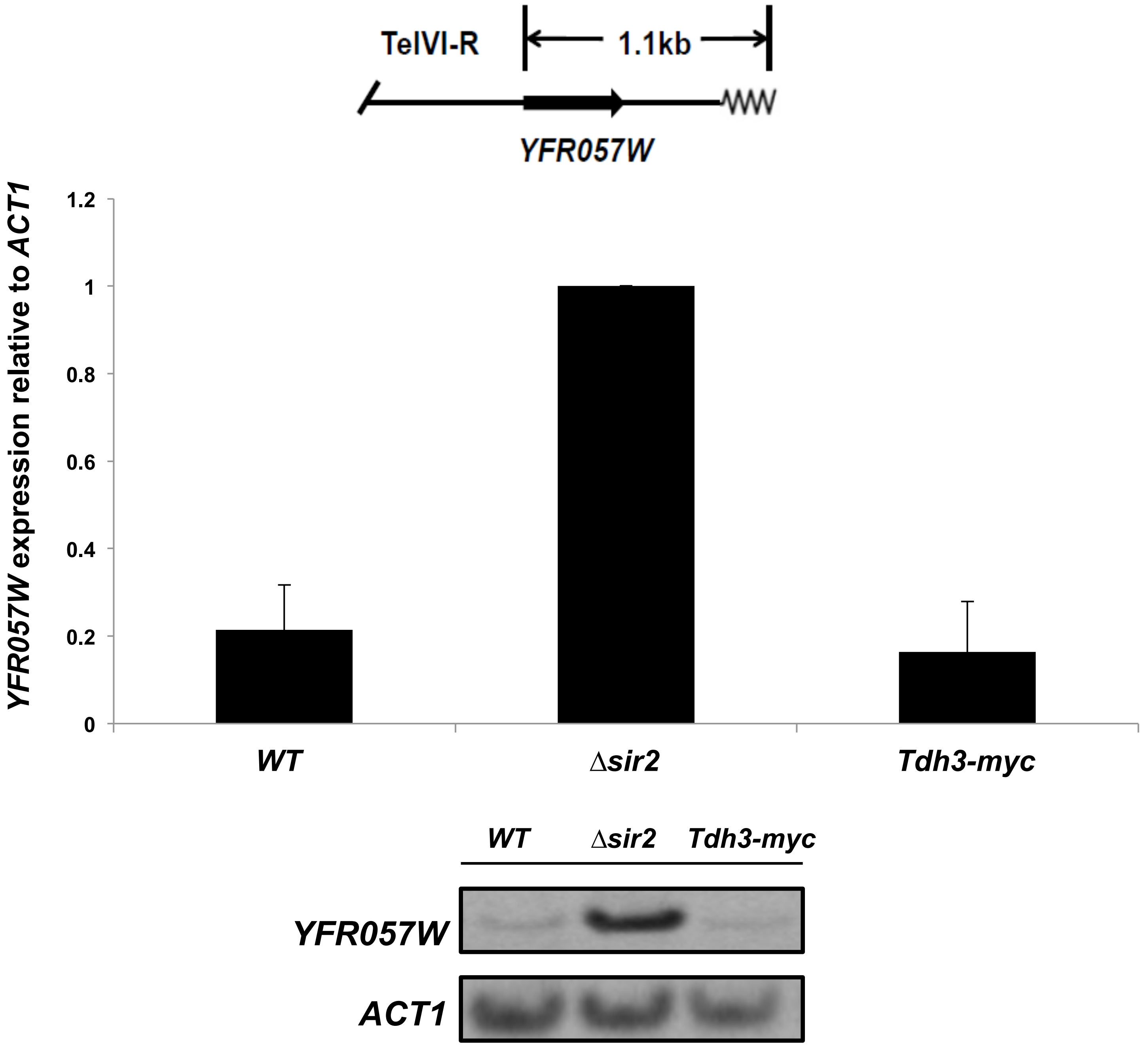

Supplementary Figure 2B

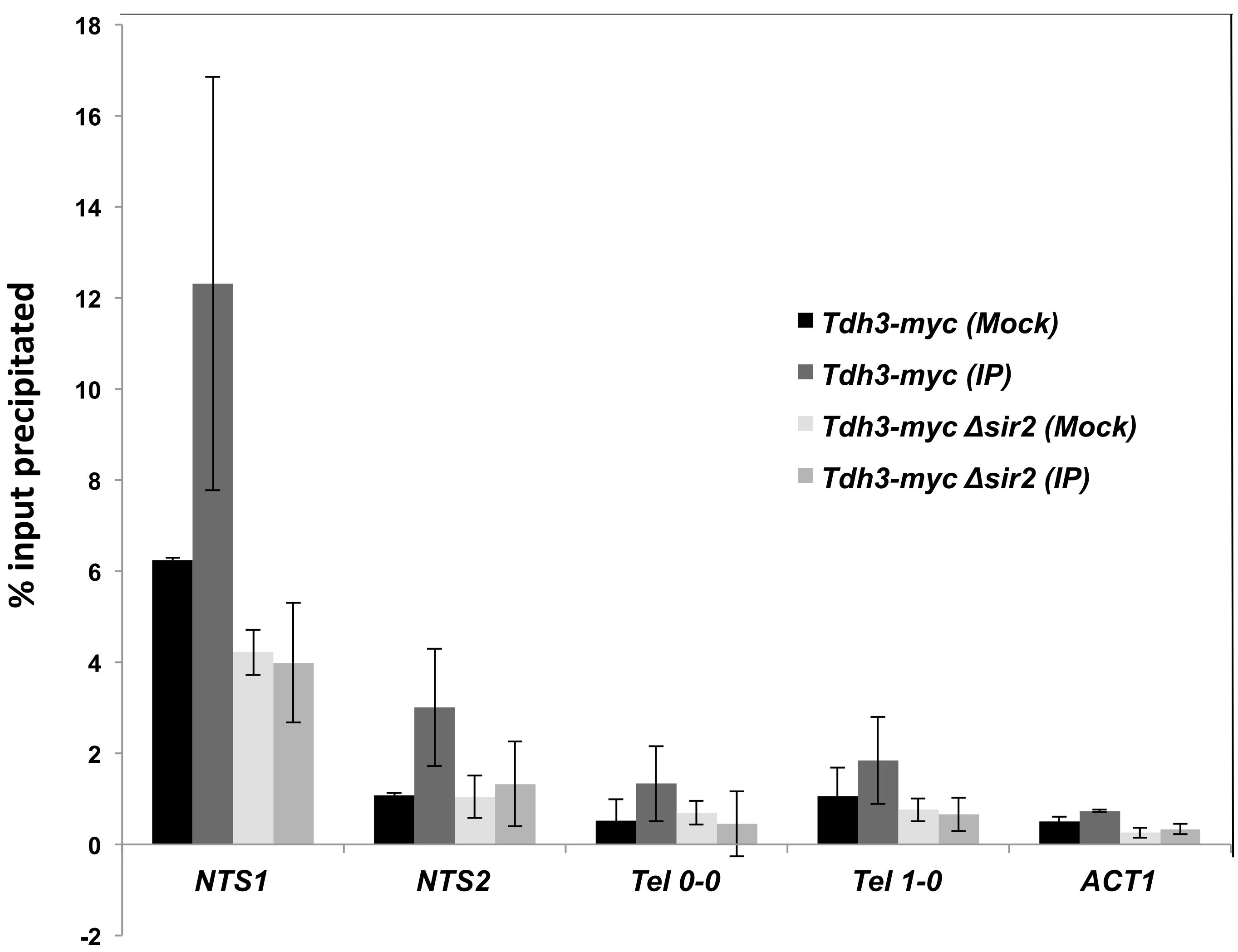

Supplementary Figure 2C

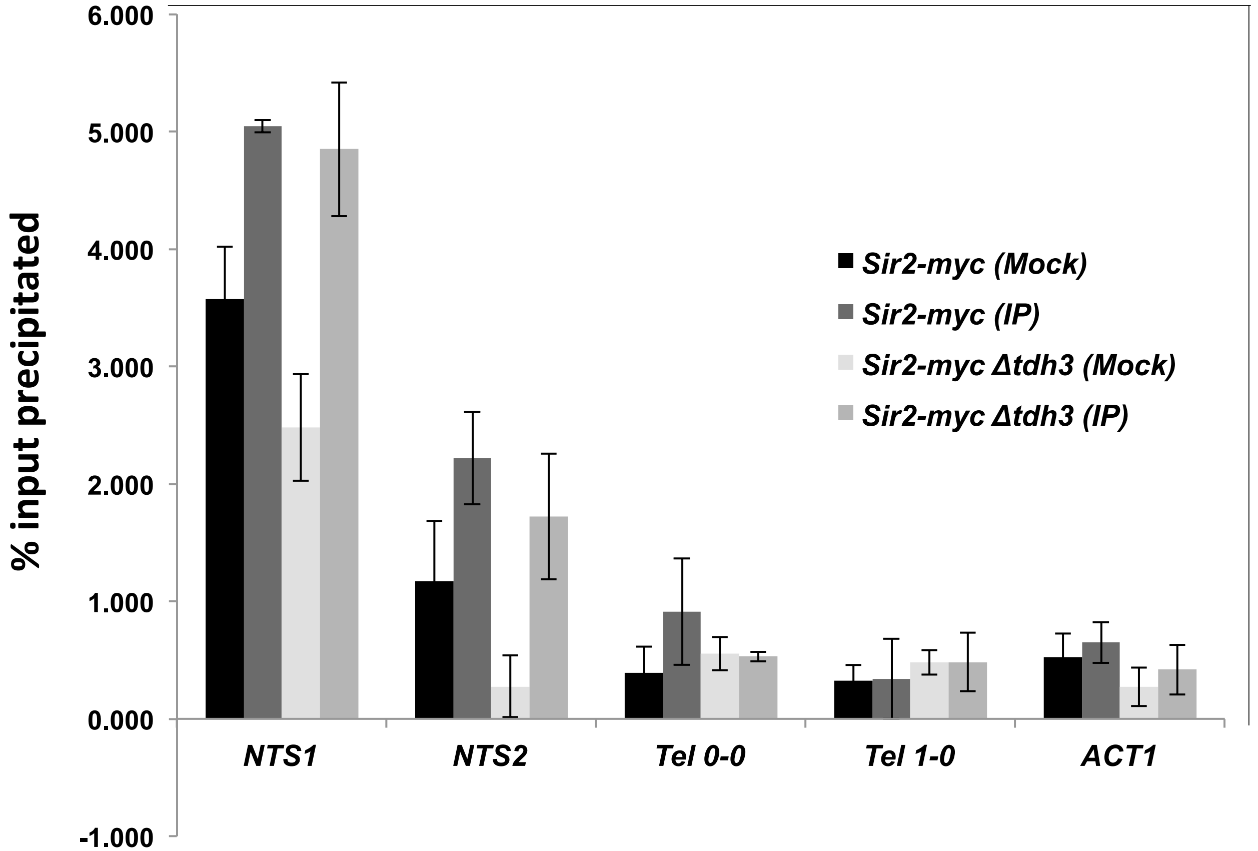

Supplementary Figure 2D

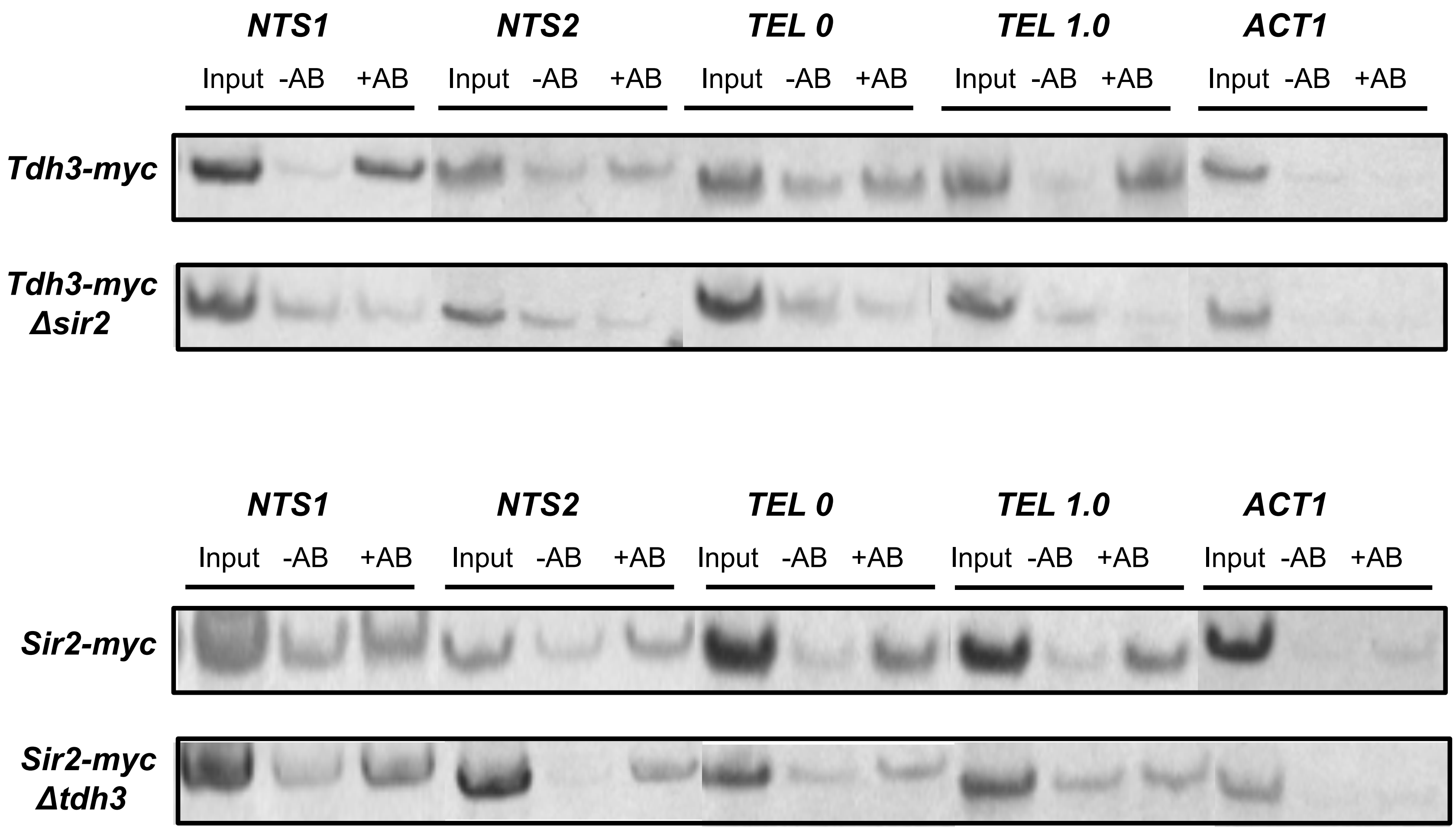

Supplementary Figure 2E

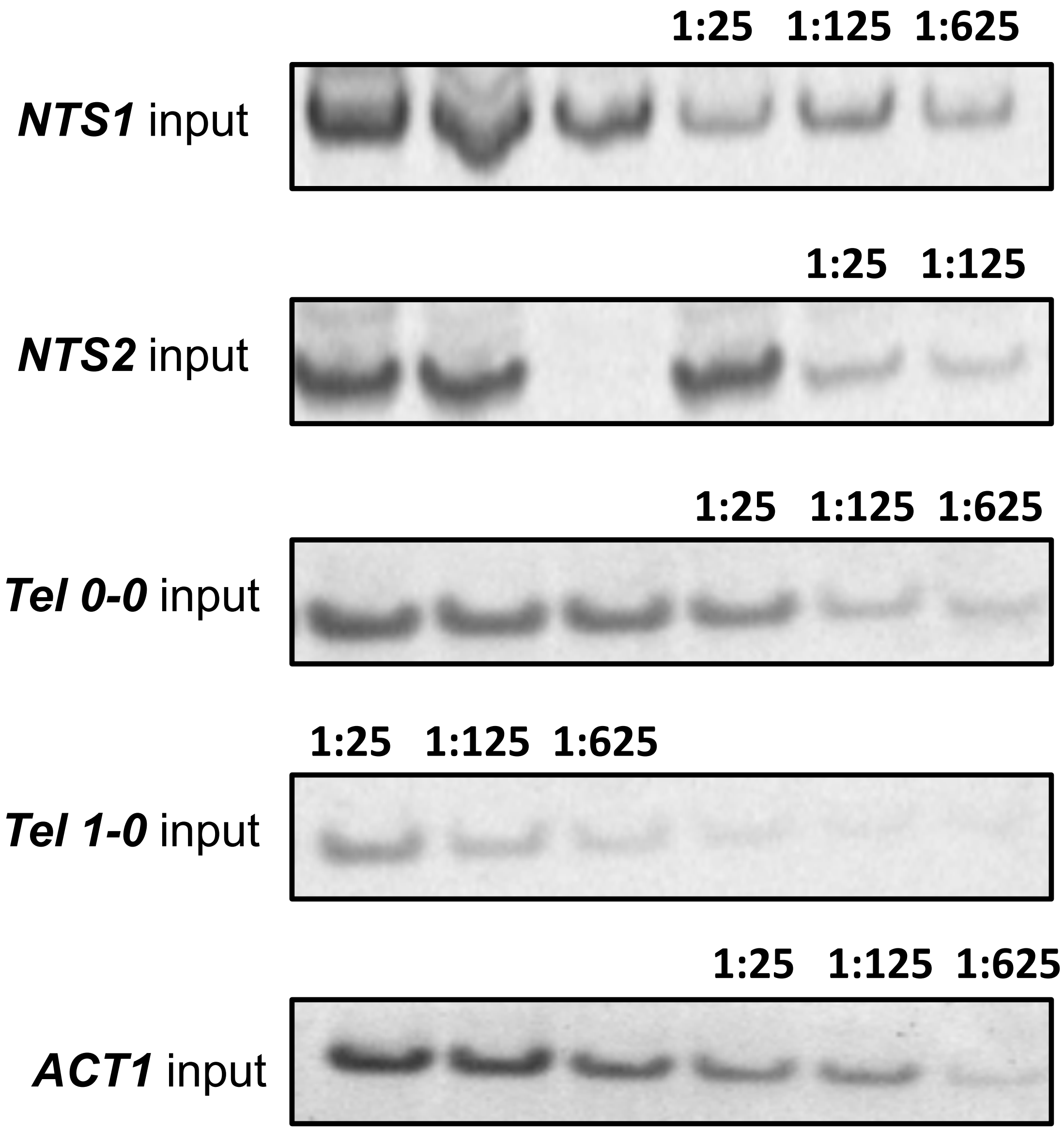

Supplement: Figure S2 — Tdh3 binds to telomeres and rDNA in a Sir2-dependent manner. (A) Cells expressing Tdh3-myc exhibit normal silencing. Expression of the native telomere-proximal gene YFR057W was examined by quantitative RT-PCR in matched strains expressing endogenous Tdh3 or Tdh3-myc. A strain lacking Sir2 was used as an unsilenced control. (B) The chromatin immunoprecipitation data presented in Figure 7 is shown, indicating the % of input DNA that was recovered for each locus. “IP” refers to the signal achieved in ChIP experiments performed with an antibody to the myc tag; “mock” indicates the signal seen in control immunoprecipitations with no antibody. Tdh3-myc association at two positions adjacent to telomere V and two positions within the rDNA repeats (NTS1 and NTS2) were assessed in SIR2 and Δsir2 strains. Enrichment of a sequence within the ACT1 open reading frame was used as a negative control. (C) The association of a Sir2-myc fusion protein at the rDNA repeats, telomere VR, and the ACT1 gene was assessed in TDH3 and Δtdh3 strains. (D) Representative experiments conducted to generate the data shown in Figures 7, S2B, and S2C are shown. Strains queried are listed on the left. Gels depict the signal after PCR from input chromatin, chromatin immunoprecipitated with an antibody to the myc tag (“+AB”) and from mock immunoprecipitations in which no antibody was used (“−AB”). (E) Representative control experiments to ensure linearity of the qPCR used for ChIP experiments are shown. PCR was performed as described in Materials and Methods using primers to the indicated loci. PCR was conducted on a dilution series of input chromatin DNA; a range of the dilution series is labeled in each panel. (PDF) [file pgen.1003871.s002.pdf]
