# Supplementary material for: Yeast Tdh3 (Glyceraldehyde 3-Phosphate Dehydrogenase) Is a Sir2-Interacting Factor That Regulates Transcriptional Silencing and rDNA Recombination
Source: PLoS Genet. 2013 Oct 17;9(10):e1003871. doi: 10.1371/journal.pgen.1003871 (PMC3798266; doi:10.1371/journal.pgen.1003871)
Supplement: Table S1 — Sequences of primers used for mRNA measurements and chromatin immunoprecipitations are shown in Supplementary Table 1. (PDF) [file pgen.1003871.s005.pdf]

Supplementary Table 1. Primer sequences

Primer	Target	Sequence
Primers for mRNA measurements		
SP 236	*ACT1*	CTGAATTAACAATGGATTCTG
SP 237	*ACT1*	CATCACCAACGTAGGAGTC
SP 1450	*YFR057W*	CTCTAACATAACTTTGATCCTTACTCG
SP 1451	*YFR057W*	CTAGTGTCTATAGTAAGTGCTCGG
Primers for chromatin immunoprecipitation experiments		
SP638	*ACT1*	ATCGTTATGTCCGGTGGTACC
SP639	*ACT1*	TGGAAGATGGAGCCAAAGC
SP892	*TEL V 0.0*	GGTGGCTCTGGAGGCTCAT
SP893	*TEL V 0.0*	CATCAACTGCACATAATTGGCG
SP886	*TEL V 1.0*	TTGTAATGACGAGCATATCGGTG
SP887	*TEL V 1.0*	CGTCAACAGTTCTTAATTTCGGGT
SP1032	*NTS 1*	CAGGACATGCCTTTGATATG
SP1033	*NTS 1*	CGCCGCGTCGCCAAAAATTT
SP1030	*NTS 2*	ATTGGTAGGAGTGTGGTGGGG
SP1031	*NTS 2*	CAACCGAAACCAAAACCAAC
